# Separating Thermodynamics from Kinetics—A New Understanding of the Transketolase Reaction

**DOI:** 10.1002/cctc.201601649

**Published:** 2017-04-13

**Authors:** Stefan R. Marsden, Lorina Gjonaj, Stephen J. Eustace, Ulf Hanefeld

**Affiliations:** ^1^ Biokatalyse, Afdeling Biotechnologie Technische Universiteit Delft van der Maasweg 9 2629HZ Delft The Netherlands

**Keywords:** aldehydes, C−C coupling, enzyme catalysis, kinetics, thermodynamics

## Abstract

Transketolase catalyzes asymmetric C−C bond formation of two highly polar compounds. Over the last 30 years, the reaction has unanimously been described in literature as irreversible because of the concomitant release of CO_2_ if using lithium hydroxypyruvate (LiHPA) as a substrate. Following the reaction over a longer period of time however, we have now found it to be initially kinetically controlled. Contrary to previous suggestions, for the non‐natural conversion of synthetically more interesting apolar substrates, the complete change of active‐site polarity is therefore not necessary. From docking studies it was revealed that water and hydrogen‐bond networks are essential for substrate binding, thus allowing aliphatic aldehydes to be converted in the charged active site of transketolase.

## Introduction

**Scheme 1:**
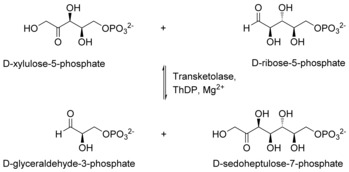
Natural TK‐catalyzed reaction.

Transketolase (TK, E.C. 2.2.1.1) is a Mg^2+^ and thiamine diphosphate (ThDP) dependent enzyme that naturally catalyzes the conversion of glycolysis‐derived metabolites into carbohydrates utilized for nucleotide synthesis and the production of essential aromatic amino acids by the Shikimate pathway.^1^ The overall reaction comprises the reversible transfer of a C_2_ ketol group and an asymmetric C−C bond formation (Scheme 1). This makes the reaction interesting for synthetic applications. A multitude of enzymatic strategies have been developed to address the substantial importance of asymmetric C−C bond formation in organic synthesis, many of which rely on the decarboxylation as driving force for the C_2_ ketol transfer.^2^, ^3^, ^4^, ^5^

**Scheme 2:**
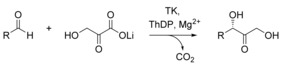
Use of LiHPA as a ketol donor in TK catalyzed synthetic applications.

To obtain an improved understanding of the TK‐catalyzed reaction, two points will be addressed herein: first, the impact of decarboxylation on the reversibility/irreversibility of the reaction and, second, the effective conversion of aliphatic substrates in TK‐catalyzed reactions although they are not the natural substrates. With regard to the first point, hydroxypyruvate (HPA) has been utilized as the ketol donor of choice because the liberation of CO_2_ results in an equilibrium constant entirely in favor of the product (Scheme 2). With this large change in Gibbs free energy, the TK‐catalyzed reaction with lithium hydroxypyruvate (LiHPA) is described as irreversible.^2^, ^3^, ^4^, ^5^, ^6^, ^7^, ^8^, ^9^, ^10^ The first *Saccharomyces cerevisiae* TK‐catalyzed synthesis of l‐erythrulose was performed with LiHPA to ensure it to be irreversible.^11^, ^12^, ^13^ However, in 2004, the TK‐catalyzed coupling of two molecules of glycolaldehyde to l‐erythrulose was reported.^14^ As the natural TK‐catalyzed reactions are reversible, irreversible product formation seems to be unlikely here. In recognition of the extensive use of decarboxylation reactions in contemporary C−C bond formation strategies, a better understanding of the actual impact of decarboxylation on the thermodynamics of TK‐catalyzed reactions is thus of great importance. In particular, the synthetically very powerful decarboxylation strategy has the disadvantage of a poor atom economy.

TKs have phosphorylated polyols as typical substrates and are naturally not disposed towards aliphatic substrates. However, as aliphatic substrates were successfully converted, it remains yet to be fully understood how this is possible. *Escherichia coli* TK has been engineered by single‐point mutations to convert a variety of aromatic and aliphatic aldehydes.^6^, ^7^ This catalytic activity is surprising because the mutations introduced in *E. coli* TK do not render the active site highly lipophilic.^6^

*S. cerevisiae* TK shares 47 % sequence identity with *E. coli* TK, and the aligned crystal structures (1QGD and 1TRK) have a root mean square deviation of 0.81 indicating extensive structural homology. Owing to its facile heterologous overexpression in *E. coli*, *S. cerevisiae* TK was chosen as model enzyme to investigate both the actual impact of decarboxylation in asymmetric C−C bond synthesis and the cause of enhanced activity towards aliphatic aldehydes previously observed for single‐point mutations.^6^, ^7^

## Results and Discussion

**Figure 1:**
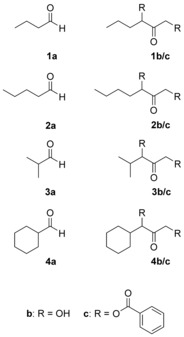
Overview of substrates (**a**), products (**b**), and derivatized products (**c**) required for chiral analysis. Products **1**–**3** (**b**) and (**c**) were obtained in the **3**‐(*S*) configuration with TK. Products **4 b/c** were not accessible enzymatically.

The *E. coli* TK mutants D469E and D469T have demonstrated that highly polar or even charged amino acids improve enzyme activity towards aliphatic aldehydes.^6^ This is in contrast to our results that showed that nonphosphorylated substrates are better converted by TK mutants of reduced polarity (R528K, R528Q, R528K/S527T, and R528Q/S527T).^15^, ^16^ Therefore, the equivalent mutations D477E and D477T were created in *S. cerevisiae* TK to allow for direct comparison. The results of the reactions with the different mutants for substrates **1**–**3 a** (Figure 1, Table 1) were in line with those reported for *E. coli* TK mutants.^6^ Again, mutant D477E was identified as the best catalyst for the conversion of aliphatic aldehydes. These data, however, do not allow the evaluation of the catalytic activity of the separate mutants for synthetic application.

**Table 1:** Isolated product yields and enantiomeric excess (*ee*) of the (*S*)‐configured enantiomer.^[a]^

	WT	D477E	D477T	R528K	R528Q	R528K/S527T	R528Q/S527T
	[%]	[%]	[%]	[%]	[%]	[%]	[%]
**1 b**	11±8 (84)	34±15 (94)	8 (n.d.)^[b]^	10±8 (81)	8±2 (77)	8±3 (73)	6±4 (66)
**2 b**	7 (91)	61±13 (90)	12±4 (84)	6±4 (82)	5±1 (87)	6±1 (68)	5±1 (82)
**3 b**	0 (n.d.)^[b]^	41±20 (99)	n.d.^[b]^ (n.d.)^[b]^	3±1 (n.d.*)* ^[b]^	0 (n.d.)^[b]^	0 (n.d.)^[b]^	0 (n.d.)^[b]^
**4 b**	0 (n.d.)^[b]^	0 (n.d.)^[b]^	n.d.^[b]^ (n.d.)^[b]^	0 (n.d.)^[b]^	0 (n.d.)^[b]^	0 (n.d.)^[b]^	0 (n.d.)^[b]^

[a] Reaction conditions: 20 U of *S. cerevisiae* TK, 5 mm ThDP, 18 mm Mg^2+^, 1 mmol LiHPA, 1 mmol aldehyde, 10 mL final volume in 5 mm sodium phosphate buffer, pH 7.0, 25 °C, 200 rpm, 18 h. Enantiomeric excess in % [b] Not determined.

Analysis of the Michaelis–Menten parameters confirmed these results. Mutant D477E performed best in the conversion of aliphatic aldehydes **1 a** and **2 a** showing an enhanced activity of 50‐ to 100‐fold compared to the WT (Table 2). Although mutations at position R528, which natively binds to the phosphate group of phosphorylated substrates,^15^, ^16^ and the incorporation of a group mutation strategy^17^ did enhance enzyme activity, the improvements were only minor compared to the effect of mutation D477E.

**Table 2:** Michaelis–Menten parameters.^[a]^

		WT	D477E	D477T	R528K	R528Q	R528K/S527T	R528Q/S527T
**1 b**	*k*_cat_ *K*_M_ *k*_cat_ *K*_M_^−1^	1.2 272 4.2	42 163 260	0.5 48 10	0.8 181 4.4	1.5 239 6.1	1.9 260 7.4	0.8 106 7.5
**2 b**	*k*_cat_ *K*_M_ *k*_cat_ *K*_M_^−1^	0.8 327 2.4	9.3 40 233	0.4 43 9.9	0.1 16 6.9	2.1 611 3.5	0.3 67 4.2	0.4 42 8.2
**3 b**	*k*_cat_ *K*_M_ *k*_cat_ *K*_M_^−1^	0.4 150 2.9	0.6 66 8.3	n.d.^[b]^	n.d.^[b]^	0.3 99 2.5	0.3 86 3.7	n.d.

[a] *k*_cat_ in s^−1^, *K*_M_ in mm, *k*_cat_ *K*_M_^−1^ in m ^−1^ s^−1^. For error bars, see Supporting Information Figures S10–S12. Reaction conditions: 50 μg purified *S. cerevisiae* TK:, 1 mm ThDP, 4 mm Mg^2+^, 100 mm LiHPA, 5–150 mm aldehyde, 5 mm sodium phosphate buffer, pH 7.0, 25 °C, 500 rpm. [b] Not determined.

### In silico docking studies

**Figure 2:**
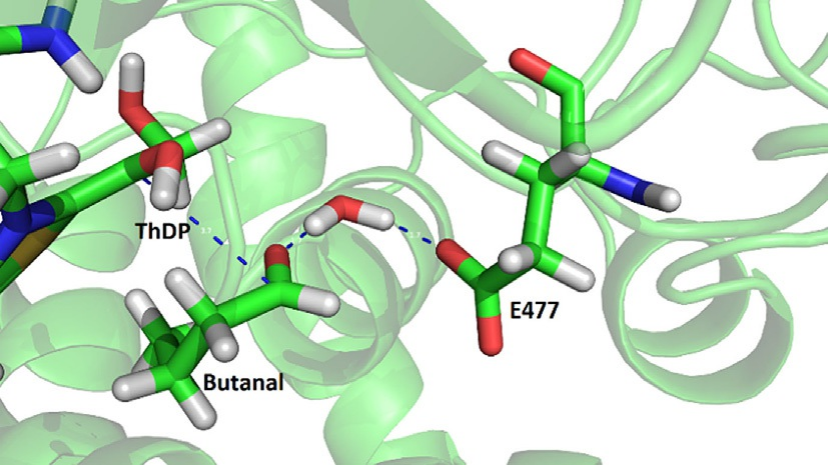
In silico docking of butanal into the energy‐minimized mutant active site D477E using YASARA program.

With an observed improvement of 50‐ to 100‐fold in *k*_cat_ *K*_M_^−1^ for the conversion of substrates **1 a** and **2 a** with D477E by only a single‐point mutation, mutation D477E was introduced in silico into the corresponding crystal structure 1GPU^18^ to investigate the resulting changes in the active site. The obtained model was energy‐minimized before docking of substrates **1 a**–**4 a** into the active site using YASARA program.^19^ The model showed that the extension of the carbon chain by mutating aspartate to glutamate newly enabled hydrogen‐bond interactions between the glutamate carboxylate and the substrate carbonyl groups bridged by a molecule of coordinated water at 1.7 Å each. In this manner, the substrate is correctly aligned towards the cofactor and the forming oxyanion is stabilized by charge delocalization during the nucleophilic attack. This interaction was correctly predicted by the model for the converted substrates **1 a**–**3 a** and not predicted for the unconverted substrate **4 a** (Figure 2 and Figure S5–S8, Supporting Information). In combination with preparative and kinetic data, the docking studies illustrate that correct substrate orientation towards the activated cofactor (improving not only *k*_cat_, but potentially also *K*_M_) is of greater importance for catalysis than an increase based solely on substrate affinity (improving only *K*_M_). This would also explain why the introduction of an isoleucine into the equivalent position in the TK of *Geobacillus stearothermophilus* did not lead to such large rate improvements.^20^

### Mechanistic reflections

**Scheme 3:**
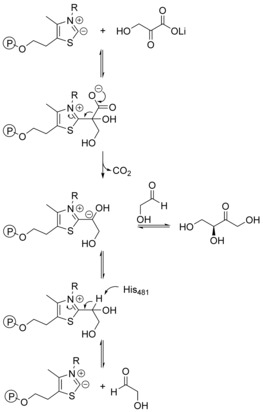
Proposed mechanism for the formation of the activated ketol bearing the carbanion by either decarboxylation (top) or catalytic deprotonation (bottom).

For the synthesis of l‐erythrulose from glycolaldehyde and LiHPA as substrates in aqueous solution under standard conditions, the total change in Gibbs free energy Δ_r_ *G* ^0^ amounts to −264.5 kJ mol^−1^ (l‐erythrulose, S18 in the Supporting Information), largely owing to the contribution of decarboxylation. Overall, this would correspond to an equilibrium constant of *K*_eq_=10^46^ in favor of the product. In 2004, the one‐substrate TK‐catalyzed reaction coupling two molecules of glycolaldehyde to l‐erythrulose was reported,^14^ and in strong contrast to the decarboxylation‐driven reaction, an equilibrium constant of *K*_eq_=5.0 was calculated from the change in Gibbs free energy (Δ_r_ *G* ^0^=4.0 kJ mol^−1^ l‐erythrulose in aqueous solution under standard conditions, S18, Supporting Information). In agreement with the natural reversible reactions, the one‐substrate reaction should, therefore, be a true equilibrium reaction. In the proposed mechanism for TK‐catalyzed reactions with LiHPA, the thermodynamically irreversible decarboxylation of LiHPA effects the direct formation of the carbanion on the activated ketol. For the one‐substrate reaction, however, the activated carbanion must be formed by catalytic deprotonation from residue His481 as an alternative to decarboxylation, generating the activated intermediate at a lower rate in comparison to its generation by decarboxylation. At the stage of the activated ketol bearing the carbanion, the enzyme can no longer distinguish whether the carbanion was formed by a reaction pathway involving decarboxylation or by catalytic deprotonation. The information about the thermodynamically driving force of decarboxylation is therefore already lost prior to the actual product formation. These mechanistic reflections consequently suggest that TK‐catalyzed synthesis reactions are reversible for the mechanism of the one‐substrate reaction, splitting the product back into one molecule of the respective acceptor aldehyde and one molecule of glycolaldehyde. The thermodynamic contribution of decarboxylation, therefore, should not affect the position of the overall equilibrium (Scheme 3) and thus makes irreversible product formation unlikely. In conclusion, it should thus be possible to avoid the release of CO_2_ and improve the atom economy of the reaction even on a preparative scale.

### Equilibrium analysis

**Scheme 4:**
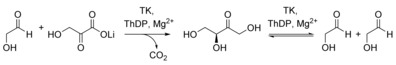
Decarboxylation‐driven reaction (left) and one‐substrate reaction (right) for the TK‐catalyzed synthesis of l‐erythrulose.

**Figure 3:**
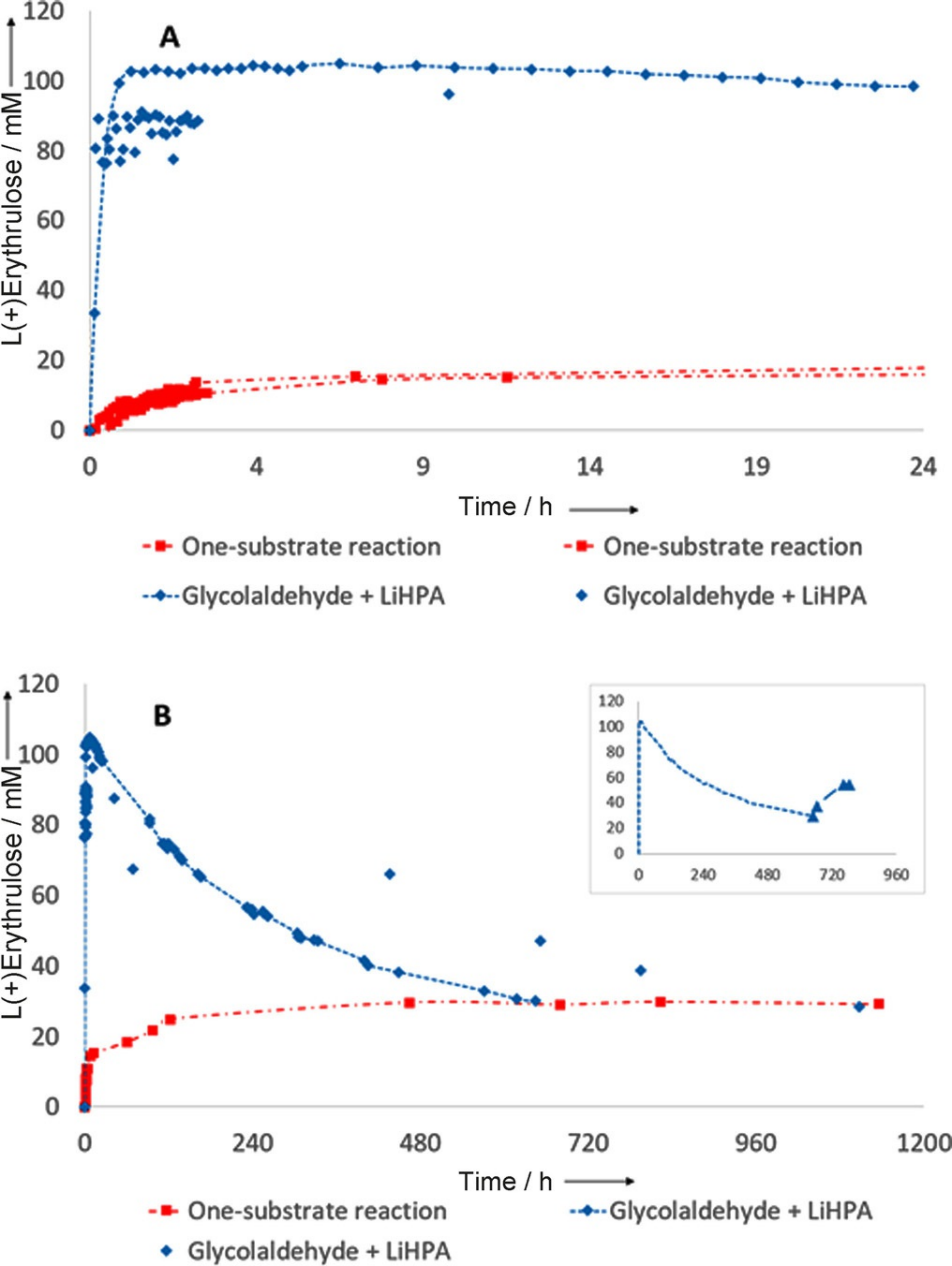
TK reaction producing l‐erythrulose as followed by ^1^H NMR analysis. 200 μg WT TK, 5 mm ThDP, 18 mm Mg^2+^, 5 mm sodium phosphate buffer pH 7.0. For the one‐substrate reaction (red), 200 mm glycolaldehyde, and for the decarboxylation‐driven reaction (blue), 100 mm glycolaldehyde and 100 mm LiHPA were used. A) Initial 24 h showing complete conversion in the decarboxylation‐driven reaction; B) Extended time course showing equilibration of both reactions towards the equilibrium concentration of 29.1±0.6 mm for erythrulose. Inset: addition of LiHPA after 650 h showing retained enzyme activity (triangles).

To confirm the theoretically suggested reversibility of the TK‐catalyzed product formation experimentally, l‐erythrulose was synthesized by both the one‐substrate reaction coupling two molecules of glycolaldehyde and the conversion of glycolaldehyde with LiHPA to afford the product l‐erythrulose in 100 mm concentration for complete conversion using wild‐type (WT) *S. cerevisiae* TK (Scheme 4). The reactions were performed in sealed NMR tubes allowing for direct measurements of the product formation of erythrulose^21^ (Figure S40). The substrates were not followed because consumption was completed within 30 min for LiHPA and because of the issue of oligomerization and hydration of glycolaldehyde in aqueous solution.^22^ Both reactions were followed over an extended period of time. In line with the results earlier published,^14^ l‐erythrulose formation was observed. The one‐substrate reaction proceeded relatively rapidly (Figure 3 A) but was limited to less than 30 % yield by the thermodynamic equilibrium of the reaction (Figure 3 B).

If LiHPA was used as ketol donor, fast and complete conversion was observed as expected^3^, ^4^, ^5^, ^6^, ^7^, ^8^, ^9^, ^10^, ^15^, ^16^, ^20^ (Figure 3 A). If this reaction was thermodynamically controlled by the release of CO_2_ it should stop at complete conversion. However, in line with a reversible reaction, a slow decline of l‐erythrulose concentration was subsequently observed ultimately coinciding with the equilibrium concentration of the one‐substrate reaction at *K*_eq_=29.1±0.6 mm. The synthesis reaction was thus shown to benefit from a kinetic effect enabling high yields at the beginning of the reaction. The reverse reaction causing thermodynamic equilibration to occur over a time course of several weeks then shifted the product distribution; in line with the outcome of the one‐substrate reaction (Figure 3 B). To confirm that the observed equilibration was indeed enzyme‐catalyzed, another portion of LiHPA was added at the end. Retained enzymatic activity was observed (Figure 3 B, inset), whereas control reactions without enzyme showed no conversion.

The representative formation of l‐erythrulose from glycolaldehyde and LiHPA was thus shown to be initially kinetically controlled contrary to all earlier assumptions about the thermodynamic driving force of CO_2_ release. The proposed reaction mechanisms depicted in Scheme 3 suggest these findings to generally hold true for all TK‐catalyzed reactions with HPA. Following the example of the pyruvate decarboxylase catalyzed synthesis of (*R*)‐phenylacetylcarbinol with acetaldehyde replacing the traditional donor substrate pyruvate,^23^ the development of novel strategies which do not rely on decarboxylation is of commercial relevance. To do so, a correct understanding of decarboxylation is of utmost importance. In syntheses in which aldehydes other than glycolaldehyde are used as acceptors, formation of the desired product will be competing with the one‐substrate reaction. Active‐site engineering as pioneered by Pohl for a range of ThDP‐dependent enzymes could ensure that glycolaldehyde will be the donor molecule in mixed carbo‐ligation reactions.^24^

## Conclusions

Creating novel interactions between an active‐site residue and a desired substrate should include a network of hydrogen bonds.^25^, ^26^, ^27^, ^28^, ^29^ As was shown, this is an effective strategy to increase the substrate's affinity towards the active site, although a polarity‐based analysis would suggest the opposite. This alternative approach for the rational mutagenesis of transketolases towards hydrophobic substrates was demonstrated. As decarboxylation‐driven C−C bond formation reactions traditionally are misinterpreted in literature as irreversible, mechanistic reflections and experimental evidence unambiguously showed the reaction to be initially under kinetic control. In the context of man‐made climate change, people thus have to extensively re‐evaluate the choice of donor substrates and the utilization of decarboxylation strategies in synthetic applications.

## Experimental Section

### Materials

Chemicals and solvents were obtained as reagent grade from Sigma–Aldrich. Aldehydes were freshly distilled and their purity confirmed by ^1^H NMR before usage. Petroleum ether (bp 40–60 °C) was freshly distilled before usage. Lithium hydroxypyruvate was obtained both commercially and synthesized as previously described.^30^

### Methods

Reaction progress was monitored by TLC (TLC Silica gel 60 F_254_, Merck) using UV light and a potassium permanganate stain for visualization. NMR spectra were recorded using an Agilent 400 MHz (^1^H, 9.4 Tesla) spectrometer operating at 399.67 MHz for ^1^H at 298 K and were subsequently interpreted using MNOVA. A benzene‐D_6_ NMR insert capillary (Sigma–Aldrich) was used for external locking during water suppression experiments using the PRESAT‐PURGE pulse sequence in sealed Wilmad screw‐cap NMR tubes (Sigma Aldrich). Spectra were recorded by using a recycle delay of 2 s and 64 repetitions. Preparative‐scale bioconversions were performed in an Excella E24 Incubator Shaker (New Brunswick Scientific).

Preparation of cell free extract: The cell pellet containing the respective mutant TK was resuspended in sodium phosphate buffer (5 mm, pH 7.0, 10 mL g^−1^ cell pellet). A protease inhibitor (PMSF, 200 μL, 0.1 m in EtOH) was added to each sample. Lysozyme was added at 20 mg g^−1^ cell pellet and a spatula tip of DNAse was added to each sample and incubated on ice for 30 min. The cells were broken using a sonifier 250 (Branson) and the cell debris removed by centrifugation.

Enzyme purification: The cell pellet was resuspended in binding buffer (5 mm sodium phosphate, pH 7.4, 20 mm imidazole) and incubated with PMSF, lysozyme and DNAse as previously described. The cells were subsequently broken using a cell disrupter (Constant Systems Ltd, 1.8 kbar), the cell debris removed by centrifugation and the cell free extract filtered (0.45 μm). Affinity chromatography was performed on a NGC Quest 10 system (Biorad) using XK16/20 columns (GE Healthcare Life Sciences) packed with 10 mL Ni‐sepharose 6 FF resin (GE Healthcare Life Sciences). For full details see Supporting Information.

Synthesis of racemic standards: Racemic standards were synthesized according to a method previously described.^31^ *N*‐methylmorpholine (330 μL, 3.0 mmol, 1.0 equiv.) was dissolved in water (40 mL) and the pH was adjusted to 8.0 using 10 % HCl. LiHPA (330 mg, 3.0 mmol, 1.0 equiv.) and the corresponding aldehyde (3.0 mmol, 1.0 equiv.) were added and the reaction was stirred overnight at room temperature. Conversion was monitored by TLC (*n*‐pentane/EtOAc 1:1). Silica powder was added, the water removed in vacuo and the crude product purified by flash chromatography (*n*‐pentane/EtOAc 1:1). For full details see ESI.

Dibenzoylation of enantiomers: Dihydroxyketone (1.0 equiv.) was dissolved in dry dichloromethane (10 mL) under N_2_ atmosphere in a flame dried round bottomed flask. Dry triethylamine (10.0 equiv.) and benzoyl chloride (5.0 equiv. per hydroxyl) were added and the reaction mixture was stirred for 2 h at room temperature. It was quenched by addition of saturated NaHCO_3_ (30 mL), the phases separated, and the organic phase was washed (sat. NaHCO_3_, 2×50 mL, then saturated NH_4_Cl, 1×50 mL, then brine, 1×30 mL). The organic phase was dried over Na_2_SO_4_, the solvent was removed in vacuo and the crude product was purified by flash chromatography for the racemic standards (petroleum ether/EtOAc 10:1). Purification by flash chromatography was omitted in the determination of the enantiomeric excess. For full details see Supporting Information.

Glycolaldehyde activity assay:^15^ The volumetric activity of cell free extracts was determined by incubating 50 μL with the cofactors (25 °C, 800 rpm, 20 min, ThDP: 5 mm, Mg^2+^: 18 mm). LiHPA and glycolaldehyde were added to achieve final concentrations of 50 mm in 300 μL total reaction volume, 5 mm sodium phosphate buffer, pH 7.0). The reaction mixture was shaken (25 °C, 800 rpm, 15 min), quenched by addition of TFA (300 μL, 0.2 % v/v), the enzyme precipitated by centrifugation and analyzed by RP HPLC (R^2^=0.998) to determine the volumetric activity. Owing to considerably varying volumetric activities of cell free extracts the enzyme content was normalized to 20 U of activity based on a glycolaldehyde activity assay previously reported.^15^

Computational docking of glycolaldehyde into the corresponding mutant active sites with YASARA predicted comparable binding energies for all mutants. It was thus concluded that none of the mutations are likely to have introduced a major bias to an activity‐based analysis using glycolaldehyde as reference. For full details, see Supporting Information.

Preparative‐scale bioconversions. Cell‐free extract (20 U based on the glycolaldehyde activity assay) was incubated with its cofactors (20 min, room temperature, 5 mm sodium phosphate buffer, pH 7.0, 18 mm ThDP, and 5 mm Mg^2+^). LiHPA (110 mg, 1.0 mmol, 1.0 equiv.) and the corresponding aldehyde (1.0 mmol, 1.0 equiv.) were added and the reaction volume was adjusted to 10 mL. The reaction was conducted in a sealed flask overnight (25 °C, 200 rpm). The product was extracted with MTBE (2x, 40 mL) and the solvent was removed in vacuo.

Chiral separation: Enantiomers were derivatized by dibenzoylation and chiral separation was performed on a Shimadzu LC‐20AD prominence system equipped with a Chiralpak AD‐H column (0.46×25 cm, Daicel) using *n*‐heptane/*i*PrOH 97:3 as mobile phase (35 °C, 1 mL min^−1^).

Analytical quantitation:^15^ Dihydroxyketone product concentrations were determined by reversed‐phase HPLC on a Shimadzu LC‐20AD prominence system equipped with an IC‐Sep Coregel 87H3 column (0.4×25 cm, Transgenomic). The absorbance was followed at 210 nm by using 0.1 % (*v*/*v*) aqueous trifluoroacetic acid (TFA) at pH 2.5 as a mobile phase (60 °C, 0.8 mL min^−1^).

Determining Michaelis–Menten parameters: Individual reaction times were initially determined to measure the parameters under credible initial rate conditions (<20 % conversion). The buffered reaction mixture (300 μL, 5 mm sodium phosphate, pH 7.0) containing holotransketolase (50 μg/337 pmol, 1 mm ThDP, 4 mm Mg^2+^), 100 mm LiHPA, and the corresponding aldehyde at varied concentrations (5–150 mm) were incubated (25 °C, 500 rpm) in duplicate. The reactions were quenched by 1:1 addition of 0.2 % (*v*/*v*) TFA, the enzyme was precipitated by centrifugation and the supernatant was subjected to reversed‐phase HPLC analysis. A Michaelis–Menten type nonlinear fit was obtained from the Excel built‐in solver successively minimizing the sum of the squared errors between measured and fitted data points converging towards values for *K*_M_ and *v*_max_. For full details see Supporting Information.

Equilibrium analysis by NMR.^21^, ^32^ The benzene signal (s, 7.15 ppm) from a NMR insert capillary was used as a reference and its integral (including ^13^C satellites) was normalized to 1000. The erythrulose concentration was followed by its characteristic peaks 4.61 (1 H, d, ^2^ *J*_HH_ 19.6 Hz), 4.52 (1 H, d, ^2^ *J*_HH_ 19.6 Hz). L(+)‐erythrulose was obtained in the highest quality commercially available (Sigma–Aldrich) and the calibration curve was corrected mathematically for a purity of 85 %. Enzyme (WT TK, 200 μg, 1.35 nmol) was incubated with its cofactors (25 °C, 20 min, ThDP: 5 mm, Mg^2+^: 18 mm, 5 mm sodium phosphate buffer pH 7.0). LiHPA‐driven conversion: glycolaldehyde and LiHPA were added to achieve final concentrations of 100 mm each and the reaction volume was adjusted to 500 μL. One‐substrate reaction: glycolaldehyde was added to achieve a final concentration of 200 mm and the reaction volume was adjusted to 500 μL.

Computational docking studies: In silico docking studies were performed with YASARA (Version 16.2.18) using the crystal structures 1TRK (free ThDP cofactor) and 1GPU (containing the activated ketol) for *S. cerevisiae* TK and 1QGD for *E. coli* TK. The simulation box was defined at 10 Å around the thiamine C2 in 1TRK and around the ylide anion in 1GPU. The substrates were energy minimized with ChemBio3D Ultra 12.0 (Cambridgesoft) using MM2 energy minimization. The mutation D477E was introduced into 1GPU and the model was subsequently energy minimized using YASARA before docking. For full details see ESI.

## Conflict of interest

*The authors declare no conflict of interest*.

## Supporting information

As a service to our authors and readers, this journal provides supporting information supplied by the authors. Such materials are peer reviewed and may be re‐organized for online delivery, but are not copy‐edited or typeset. Technical support issues arising from supporting information (other than missing files) should be addressed to the authors.

Supplementary
