# Supplementary material for: Separating Thermodynamics from Kinetics—A New Understanding of the Transketolase Reaction
Source: ChemCatChem. 2017 Apr 13;9(10):1808–14. doi: 10.1002/cctc.201601649 (PMC5573996; doi:10.1002/cctc.201601649)
Supplement: Supplementary file 1 — Supplementary [file CCTC-9-1808-s001.pdf]

Heterogeneous & Homogeneous & Bio- & Nano-
CHEMCATCHEM
CATALYSIS

Supporting Information

Separating Thermodynamics from Kinetics—A New Understanding of the Transketolase Reaction

Stefan R. Marsden, Lorina Gjonaj, Stephen J. Eustace, and Ulf Hanefeld*[a]

cctc_201601649_sm_miscellaneous_information.pdf

Contents

1 Materials and Methods **1**
1.1 Molecular Biology and Enzyme Expression 1
1.1.1 Stock Solutions 2
1.1.2 Protocols 3
1.2 Chemistry and Biotransformations 8
1.2.1 Lithium Hydroxypyruvate Synthesis 10
1.2.2 Synthesis of Racemic Standards 11
1.2.3 Dibenzoylation Procedure for Racemic Standards . . . 12
1.2.4 Glycolaldehyde Activity Assay 13
1.2.5 Preparative Scale Bioconversions 13
1.2.6 Determination of Michaelis-Menten Parameters 14
1.2.7 Equilibrium Analysis by NMR 15
1.3 Modelling and *in silico* Methods 16
1.3.1 Thermodynamic Model 16
1.3.2 Computational Docking Studies with YASARA 18

2 References **21**

3 Appendix **22**
3.1 Protein purification 22
3.2 Chiral Analysis by HPLC 23
3.3 Gene and Primer Sequences 26
3.4 Michaelis-Menten Curve Fits 28
3.5 Thermodynamic Model Source Code 31
3.6 NMR Spectra 33

1 Materials and Methods

1.1 Molecular Biology and Enzyme Expression

Bacterial work was performed under sterile conditions using autoclaved consumables and solutions under a bunsen burner flame. Cells were broken using a Sonifier 250 (Branson) for volumes up to 5 mL of cell suspension, or a Multishot Cell Disrupter (Constant Systems Ltd) for larger volumes. Enzymes were purified on a NGC Quest 10 system (Biorad) using XK16/20

columns (GE Healthcare Life Sciences) packed with Ni Sepharose 6 FF resin (GE Healthcare Life Sciences). Analytical quantities of enzyme were purified using Ni-NTA spin columns (Qiagen). Protein overexpression and purity were analysed by SDS-PAGE on a PhastSystem Separation and Control Unit (Pharmacia) using Phastgel Gradient 10-15% precast gels (GE Healthcare Bio-Sciences). Protein concentrations were determined using a Bicinchoninic Acid (BCA) assay kit (Sigma-Aldrich) following the absorbance at 562 nm on a Ultrospec 2100 pro UV/Vis spectrometer (Pharmacia). Wild type Transketolase from *S. cerevisiae* (EC 2.2.1.1, gene accession number P23254) was previously cloned into the pBAD/His A plasmid adding an N-terminal His-tag to the protein. $CaCl_2$ competent cells (*E. coli* top10, Invitrogen) were used as hosts for cloning and corresponding mutants as previously reported[1]. Plasmids were isolated using a QIAprep Spin Miniprep Kit (QIAGEN) and DNA concentrations were determined using a ND-1000 Spectrophotometer (Thermo Fisher Scientific). A PCR Master Mix (2x) was bought from Thermo Fisher Scientific (K0171) and the reaction was carried out in a Tgradient PCR machine (Biometra Westburg). SYBR Safe stain (Thermo Fisher Scientific) was used for staining of DNA gels and SimplyBlue Safe stain (Invitrogen) was used for staining protein gels.

1.1.1 Stock Solutions

- Growth medium: 10 g tryptone, 10 g NaCl and 5 g yeast extract were dissolved in 1 L of milli-Q and autoclaved the same day
- 1000x Ampicillin: the ampicillin sodium salt was dissolved at 100 mg/mL in sterile milli-Q and was kept at -20°C for max. 1 week
- 20% (w/v)arabinose stock: 400 mg were dissolved in 2 mL of sterile milli-Q
- 20 mM sodium phosphate buffer, pH=7.0: 1.32 g NaH_2PO_4•$2H_2O$ and 4.131 g NaH_2PO_4•$12H_2O$ were dissolved in 1 L milli-Q
- 5 mM sodium phosphate buffer, pH=7.0 330 mg NaH_2PO_4•2 H_2O + 1.032 g Na_2HPO_4•12 H_2O in 1 L milli-Q
- 0.1M PMSF: 1.74 g phenylmethylsulfonylfluoride were dissolved in 100 mL of EtOH (absolute) and stored at -20°C for max. 1 week
- NP-10: 50 mM NaH_2PO_4, 300 mM NaCl, 10 mM imidazole, pH=8.0
- NP-20: 50 mM NaH_2PO_4, 300 mM NaCl, 20 mM imidazole, pH=8.0

- NP-500: 50 mM NaH_2PO_4, 300 mM NaCl, 500 mM imidazole, pH=8.0
- Ni-NTA binding buffer 5 mM:
  20 mM sodium phosphate, 0.5 M NaCl, 5 mM imidazole, pH = 7.4
  704 mg NaH_2PO_4•2 H_2O + 6.9 g Na_2HPO_4•12 H_2O + 29.2 g NaCl + 0.34 g imidazole were dissolved in milli-Q and the pH was adjusted to 7.4 with 6M HCl to a final volume of 1 L
- Ni-NTA binding buffer 20 mM:
  20 mM sodium phosphate, 0.5 M NaCl, 20 mM imidazole, pH = 7.4
  704 mg NaH_2PO_4•2 H_2O + 6.9 g Na_2HPO_4•12 H_2O + 29.2 g NaCl + 1.36 g imidazole were dissolved in milli-Q and the pH was adjusted to 7.4 with 6M HCl to a final volume of 1 L
- Ni-NTA elution buffer 500 mM:
  20 mM sodium phosphate, 0.5 M NaCl, 20 mM imidazole, pH = 7.4
  704 mg NaH_2PO_4•2 H_2O + 6.9 g Na_2HPO_4•12 H_2O + 29.2 g NaCl + 34 g imidazole were dissolved in milli-Q and the pH was adjusted to 7.4 with 6M HCl to a final volume of 1 L
- 1kb DNA ladder: 1 µL DNA Ladder + 1 µL 6x Loading Dye + 4 µL milli-Q Bands: 10k, 8k, 6k, 5k, 4k, 3.5k, 3k, 2.5k, 2k, 1.5k, 1k, 750, 500, 250 bp
- Low molecular weight protein ladder:
  Bands: 669 kDa, 440 kDa, 250 kDa, 140 kDa, 66 kDa
- Transketolase cofactors: 90 mM Mg^{2+}, 25 mM ThDP
  183 mg $MgCl_2$•$6H_2O$ + 115 mg thiamin diphosphate chloride were dissolved in 5 mM sodium phosphate buffer and the pH was adjusted to 7.0 using 0.1M NaOH to afford a final volume of 10 mL
- Permanganate stain: 1.5 g $KMnO_4$ + 10 g K_2CO_3 and 1.25 mL 10% NaOH were dissolved in water and filled up to 200 mL

1.1.2 Protocols

Ampicillin plates (20x) were made by carefully boiling 400 mL Luria Bertani (LB) agar in the microwave until complete dissolution. It was allowed to cool down to approximately 40°C before ampicillin was added (400 µL, 1000x). The plates were allowed to solidify under the flame before the lid was closed. Plates were stored in the fridge for max. 2 weeks.

Cryostocks were prepared from freshly grown 5 mL overnight cultures by pelleting 1.5 mL thereof, decanting the supernatant, resuspending the cell pellet in 0.5 mL of fresh LB and subsequent addition of 0.5 mL 80% glycerol. The suspensions were mixed by vortex, transferred into cryotubes and were stored at -80°C.

Transformation of plasmid DNA into $CaCl_2$ competent cells (*E. coli* top 10, Invitrogen) was performed by thawing the cells on ice for 30 minutes. 100-200 ng of plasmid DNA was then added and incubated for 30 minutes on ice. The cells were subsequently exposed to a heat shock at 42 °C for 90 seconds. They were placed directly on ice again for 2 minutes. LB (800 µL) was added and incubated at 37 °C for 1h. The cells were pelleted (13500 rpm, 5 minutes), the supernatant removed and the cells resuspended in 100 µL LB. The suspension was plated on ampicillin plates and incubated overnight at 37 °C.

Introduction of point mutations by PCR: creating the D477E mutant.

Temp.	Time	Cycles	Composition	Volume	Details
95 °C	2’		PCR Master Mix 2x	250 µL	
95 °C	10 s	16x	Plasmid DNA	12.5 µL	80 ng/µL
56-69 °C	30 s	16x	DMSO	25 µL	
72 °C	6’ 30 s	16x	Forward primer	40 µL	50 ng/µL
72 °C	10’		Reverse primer	40 µL	50 ng/µL
4 °C	∞		milli-Q	132.5 µL	
				500 µL	

Table S 1: PCR mix & programme

The mixture was distributed over ten PCR tubes and amplified using a gradient over the temperature range of 56 to 69°C. 30 µL of the product mixtures were each digested with DpnI (0.5 µL, 1h, 37 °C). Of this, 15 µL were transformed and successful point mutation was confirmed by sequencing.

Enzyme overexpression: Pre-cultures were grown overnight from single colonies (100 mL LB, 100 µg/mL ampicillin, 37 °C, 180 rpm). The inoculum was then added to 1 L of sterilised growth medium in a 5 L Erlenmeyer flask and grown until an OD_{600} of 0.6-0.8 was obtained. The cells were then induced by addition of arabinose at a final concentration of 0.02% (w/v). Enzyme overexpression was carried out overnight (37°C, 180 rpm). The cells were harvested (10'000 rpm, 10 minutes).

a) For preparation of cell free extract:
The pellet was resuspended (20 mM sodium phosphate buffer, pH=7.0). The suspension was transferred into 50 mL tubes and was centrifuged again (5'000 rpm, 10 minutes). The supernatant was discarded and the pellet was stored at -80°C.

b) For enzyme purification by affinity chromatography:
The pellet was resuspended (Ni-NTA binding buffer) and lysosyme at 20 mg/g cell pellet was added and a spatula tip of DNAse.

Preparation of cell free extract: The cell pellets containing the overexpressed enzymes were thawed on ice (30 minutes). The cells were resuspended in sodium phosphate buffer (5 mM, pH=7.0, 10 mL/g cell pellet). A protease inhibitor (PMSF, 200 µL, 0.1M in EtOH) was added to each sample. Lysosyme was added at a concentration of 20 mg/g cell pellet and a spatula tip of DNAse was added to each sample. The obtained suspension was incubated on ice for another 30 minutes. The cells were then sonicated on ice (30% duty cycle, output = 3, 4-5 minutes). The samples were centrifuged (5000 rpm, 20 minutes, 4 °C) and the supernatant stored in aliquots of 4 mL at -20 °C.

SDS-PAGE: 5 µL of protein solution was boiled with 15 µL of bromophenol blue loading dye at 90 °C for one minute. Of this solution, 2 µL were then loaded on a 10-15% gradient gel and separated. The gel was subsequently stained by incubation overnight with an aqueous solution of simply blue safe stain (100 rpm, room temperature).

Native-PAGE:
2 µL protein solution were directly loaded on a 10-15% gel and separated. The gel was subsequently stained by incubation overnight with an aqueous solution of simply blue safe stain (100 rpm, room temperature).

Protein concentrations were determined by performing the bicinchoninic

acid assay (BCA) according to the protocol provided: a working solution was prepared by mixing the bicinchoninic acid stock solution with a 4% (w/v) solution of $CuSO_4 \bullet H_2O$ in a ratio of 50:1. For the analysis, 2 mL of working solution were mixed with 100 µL of protein solution, incubated at 37°C for 30 minutes and the absorbance measured in technical duplicate at 562nm for each sample. A calibration curve was made each time using BSA as standard at concentrations of 0-1000 µg/L in intervals of 200 µg/L in biological and technical duplicate ($R^2 = 0.997$). Protein samples were measured in biological and technical duplicate.

Enzyme purification of analytical quantities: The Ni-NTA spin columns were equilibrated (600 µL NP-10, 2900 rpm, 2 minutes). The CFE was filtered (Whatman filter, 0.45 µm pore size) and charged on the spin columns (600 µL, 1600 rpm, 8 minutes, 2x). The column was washed (600 µL NP-20, 2900 rpm, 2 minutes) and the protein eluted (500 µL NP-500, 2900 rpm, 2 minutes). The column was then washed (500 µL NP-500, 500 µL H_2O (2x) and EtOH (20%)). The columns were stored in 20% EtOH in the fridge to be reused. The eluent was dialysed (1 L 5 mM sodium phosphate buffer pH=7.0, ratio 1:2000, 2x, 4 °C for 4 hours/overnight). The purified enzyme was stored in the freezer.

Preparative scale enzyme purification: The mixture of cells, lysosyme, DNAse and 1 mM PMSF in binding buffer (5 mM imidazole for R528K, R528Q, R528K/S527T, R528Q/S527T and 20 mM imidazole for WT, D477E and D477T) was incubated on ice for one hour. The cells were subsequently broken using a cell disrupter at 1.8 kbar. The cell debris was removed by centrifugation (10'000 rpm, 10 minutes, 4 °C), the supernatant filtered (Whatman filter, 0.45 µm pore size) and charged on 10 mL of Ni-sepharose 6 Fast Flow resin. Fractions were stored on ice and the peaks analysed by SDS-PAGE.

Method for WT, D477E, D477T:
Flow rate (2 mL/min.), 20 mM imidazole binding buffer. Equilibration (3 column volumes (CV), binding buffer), sample application (individual volume), column wash (3CV, binding buffer), elution (0-60% mixture with 500 mM imidazole elution buffer, 10CV, then 100%, 1CV), column wash (2CV, binding buffer).

Method for R528K, R528Q, R528K/S527T, R528Q/S527T:
Flow rate (2 mL/min.), 5 mM binding buffer. Equilibration (3CV, binding buffer), sample application (individual volume), column wash (3CV, binding buffer), elution (20-25% mixture with 500 mM imidazole elution buffer, 6CV,

then 25-60% elution buffer for 6CV, then 100% elution buffer for 1CV), column wash (2CV, binding buffer).

Combined fractions (30-40 mL respectively) were dialysed against 5 mM sodium phosphate buffer (2x, 2L, pH = 7.0, overnight).

DNA gel electrophoresis to analyse PCR products: 500 mg agarose was dissolved in 50 mL 1x TAE buffer (1% w/v) and was added 5 µL 10000x SYBR SAFE stain. The gel was allowed to solidify at room temperature and a mixture of 10 µL PCR product and 2 µL of 6x Loading Dye was loaded on the gel. A DNA ladder was added as reference and the gel was analysed (50 minutes, 100 V).

1.2 Chemistry and Biotransformations

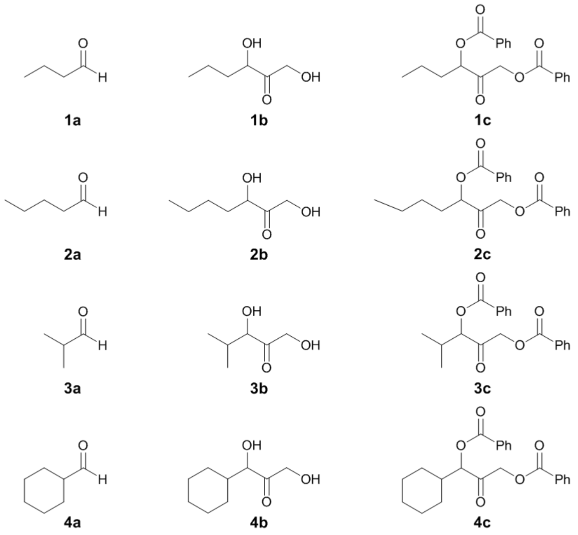

Figure S 1: Substrate aldehydes (a), products (b) and derivatised products (c). (*S*) configuration is obtained at C3 for TK catalysed reactions.

Chemicals and solvents were obtained as reagent grade from Sigma-Aldrich. Aldehydes were freshly distilled and their purity confirmed by ^{1}H NMR before usage. Petrolether (boiling point 40-60°C) was freshly distilled before usage. Lithium hydroxypyruvate was both obtained commercially and synthesised as previously described[2]. Reaction progress was monitored by TLC (TLC Silica gel 60 F_{254}, Merck) using UV light and a potassium permanganate stain for visualisation. NMR spectra were recorded using an Agilent 400 MHz (^{1}H, 9.4 Tesla) spectrometer at 298K and were subsequently interpreted using MestReC. A benzene-D_6 NMR insert capillary (Sigma-Aldrich) was used for external locking during water suppression experiments using the PRESAT pulse sequence. Preparative scale bioconversions were carried out in an Excella E24 Incubator Shaker (New Brunswick Scientific). Optical rotations were measured on a Model 343 Polarimeter (PerkinElmer).

Analytical quantitation of products containing the dihydroxyketone motif was accomplished by reversed-phase chromatography using calibration curves on a Shimadzu LC-20AD prominence system equipped with an ICSep Coregel 87H3 column (0.4x25 cm, Transgenomic). The absorbance was followed at 210 nm using 0.1% (v/v) aqueous trifluoroacetic acid pH = 2.5 as mobile phase (60°C, 0.8 mL/min).

Chiral separation of the dibenzoylated enantiomers for the analysis of enantiomeric excess was determined on a Shimadzu LC-20AD prominence system equipped with a Chiralpak AD-H column (0.46x25 cm, Daicel) using n-heptane/i-PrOH 97:3 as mobile phase (35°C, 1 mL/min).

The **Optical rotation** of commercial L(+) Erythrulose was measured at 589 nm (sodium D line) in aqueous solution (200 mg/10 mL) at 20°C, the value processed according to equation (1) and the enantiomeric excess was determined according to equation (2).

$$[\alpha]_D = \frac{100 \cdot \alpha}{1dm \cdot c} \tag{1}$$

$$ee = \frac{[\alpha]_{observed}}{[\alpha]_{max}} \cdot 100\% \tag{2}$$

1.2.1 Lithium Hydroxypyruvate Synthesis

Bromopyruvic acid (30g, 180 mmol, 1.0 eq.) was dissolved in demi water (150 mL). The pH was adjusted to 9 using 5 M LiOH. The reaction was then continued in a pH stat using 1 M LiOH overnight to maintain a constant pH of 9 over the course of the reaction. The reaction mixture was then acidified to pH = 5 using glacial acetic acid. The solvent was reduced *in vacuo* until precipitation was initialised. The product was precipitated overnight in the fridge. It was filtered, washed with ice cold water (2x) and dried in a desiccator over silica. The product was obtained as a fine white powder (8.8g, 45%) [2] and was confirmed by ^{1}H NMR in agreement with the commercial product. Both the synthesised and commercially obtained lithium hydroxypyruvate still contained traces of the starting material.

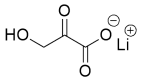

^{1}H NMR (400MHz; D_2O) δ 4.70 (2H, s, CH_2)
^{13}C NMR (100MHz; D_2O) δ 202.5 (C2), 167.0 (C1), 66.0 (C3)

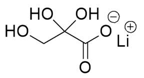

^{1}H NMR (400MHz; D_2O) δ 3.62 (2H, s, CH_2)
^{13}C NMR (100MHz; D_2O) δ 175.0 (C1), 94.3 (C2), 65.3 (C3)

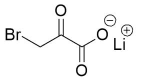

^{1}H NMR (400MHz; D_2O) δ 4.56 (0.36H, s, CH_2)
^{13}C NMR (100MHz; D_2O) δ 196.0 (C2), 169.0 (C1), 34.1 (C3)

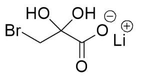

^{1}H NMR (400MHz; D_2O) δ 3.66 (1.3H, s, CH_2)
^{13}C NMR (100MHz; D_2O) δ 175 (C2), 93.5 (C1), 36.8 (C3)

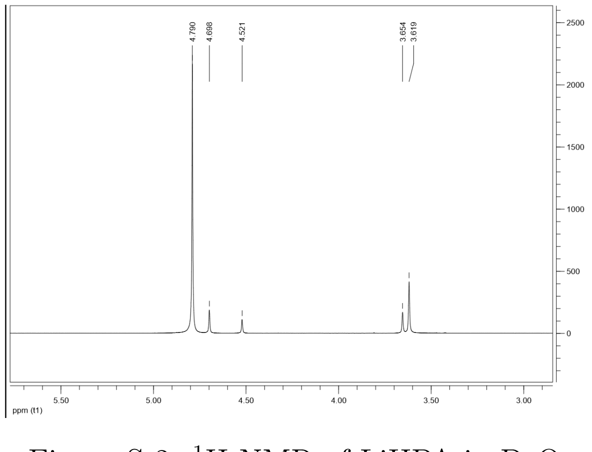

Figure S 2: ^{1}H-NMR of LiHPA in D_2O.

1.2.2 Synthesis of Racemic Standards

Racemic standards were synthesised according to a method previously described[3]. *N*-methylmorpholine (330 µL, 3.0 mmol, 1.0 eq.) was dissolved in water (40 mL) and the pH was adjusted to 8.0 using 10% HCl. LiHPA (330 mg, 3.0 mmol, 1.0 eq.) and the corresponding aldehyde (3.0 mmol, 1.0 eq.) were added and the reaction was stirred overnight at room temperature under N_2 atmosphere. The conversion of the aldehyde was monitored by TLC (*n*-pentane / EtOAc 1:1). Silica powder was added, the water removed *in vacuo* and the crude product purified by flash chromatography (*n*-pentane / EtOAc 1:1).

1b 1,3-dihydroxy-hexan-2-one:
^{1}H NMR (400MHz; $CDCl_3$) δ 4.49 (1H, d, ^{2}J 19.2Hz, C*H*HOH), 4.39 (1H, d, ^{2}J 19.2Hz, CH*H*OH), 4.31 (1H, m, C*H*OH), 1.75 (1H, m, C*H*), 1.60-1.40 (3H, m, C*H*), 0.96 (3H, t, $^3J_{HH}$ 7.2 Hz, CH_3). ^{13}C NMR (100MHz; $CDCl_3$) δ 211.6 (C2), 74.7 (C3), 65.4 (C1), 36.3 (C4), 18.0 (C5), 13.7 (C6) in agreement with literature[3]. EtOAc (4.12 (q), 2.05 (s), 1.26 (t)). The product was isolated as a colourless oil and solidified in the fridge to a white solid (78 mg / 20%). RP HPLC retention time: 13.5 min.

2b 1,3-dihydroxy-heptan-2-one:
^{1}H NMR (400MHz; $CDCl_3$) δ 4.49 (1H, d, ^{2}J 19.2Hz, C*H*HOH), 4.38 (1H, d, ^{2}J 19.2Hz, CH*H*OH), 4.30 (1H, m, C*H*OH), 2.60 (br, O*H*), 1.80 (1H, m, CH_2), 1.60 (1H, m, CH_2), 1.50-1.30 (4H, m, CH_2), 0.91 (3H, t, $^3J_{HH}$ 7.2 Hz, CH_3). EtOAc (4.12 (q), 2.05 (s), 1.26 (t)). ^{13}C NMR (100MHz; $CDCl_3$) δ 211.6 (C2), 74.9 (C3), 65.5 (C1), 33.9 (C4), 26.7 (C5), 22.4 (C6), 13.8 (C7) in agreement with literature[3]. The product was isolated as a colourless oil and solidified in the fridge to a white solid (99 mg / 23%). RP HPLC retention time: 17.3 min.

3b 1,3-dihydroxy-4-methylpentan-2-one:
^{1}H NMR (400MHz; $CDCl_3$) δ 4.46 (1H, d, ^{2}J 19.2Hz, C*H*HOH), 4.35 (1H, d, ^{2}J 19.2Hz, CH*H*OH), 4.17 (1H, m, C*H*OH), 3.1(2H, s, O*H*), 2.04 (1H, m, C*H*), 1.07 (3H, d, ^{2}J 7.2Hz, CH_3), 0.8 (3H, d, ^{2}J 6.8Hz, CH_3). ^{13}C NMR (100MHz; $CDCl_3$) δ 211.67 (C2), 79.23 (C3), 66.04 (C1), 32.08 (C4), 19.3, 15.13 (C5, C6) in agreement with literature[4]. The product was isolated as a colourless oil and solidified in the fridge to a white solid (39 mg / 8%). RP HPLC retention time: 12.5 min.

4b 1-cyclohexyl-1,3-dihydroxypropan-2-one:
Literature values[3] are given in **bold** for comparison.
^{1}H NMR (400MHz; $CDCl_3$) δ 4.48 (**4.45**)(1H, dd, ^{2}J 19.2Hz, ^{4}J 4.8Hz C*H*HOH), 4.36 (**4.34**)(1H, dd, ^{2}J 19.2Hz, ^{4}J 4.8Hz CH*H*OH), 4.17 (**3.96**)(1H,

dd, ^{3}J 5.2Hz, ^{4}J 2.8Hz C*H*OH), 2.92 (1H, t, ^{3}J 5.2Hz, CH_2OH), 2.82 (1H, d, ^{3}J 5.6Hz, CHO*H*), 1.8-1.6 (**1.87-1.45**)and 1.4-1.2 (**1.30-1.16**)(11H, m, CH_2 and C*H*).^{13}C NMR (100MHz; $CDCl_3$) δ 211.5 (**214.3**)(C-2), 79.2 (**80.7**)(C3), 66.1 (**67.4**)(C1), 42.2 (**43.0**)(C4), 29.6 (**30.3**)(C7), 26.3, 25.9, 25.8, 25.5 (**27.7, 27.3, superimposed**)(C5, C6). Hydroxyls were confirmed by H-D exchange in D_2O. The product was isolated as a white solid ($\approx$ 40 mg / 8%). RP HPLC retention time: 25.4 min.

1.2.3 Dibenzoylation Procedure for Racemic Standards

Racemic standards were dibenzoylated for chiral analysis using a general method: The dihydroxyketone substrate (1.0 eq.) was dissolved in dry dichloromethane (10 mL) under N_2 atmosphere in a flame dried round bottomed flask. Dry triethylamine (10.0 eq.) and benzoyl chloride (5.0 eq. per hydroxyl) were added and the reaction mixture was stirred for 2 hours at room temperature. The reaction was quenched by addition of saturated sodium bicarbonate solution (30 mL) and stirred for another 30 minutes. The phases were separated and the organic phase was washed (sat. $NaHCO_3$, 2x, 50 mL, sat. NH_4Cl, 1x, 50 mL, brine, 1x, 30 mL). The organic phase was dried over sodium sulphate, the solvent was removed *in vacuo* and the product purified by flash chromatography (petrolether / EtOAc 10:1). Purification by flash was omitted in the determination of the enantiomeric excess.

1c 2-oxohexane-1,3-diyl dibenzoate:
^{1}H NMR (400MHz; $CDCl_3$) δ 8.18-8.07 (4H, m, CH_{ortho}), 7.70-7.43 (6H, m, CH_{meta}, CH_{para}), 5.47 (1H, m, C*H*OR), 5.22 (1H, d, $^2J_{HH}$ 17.2 Hz, C*H*HOR), 5.06 (1H, d, $^2J_{HH}$ 17.2 Hz, CH*H*OR), 2.02 (2H, m, CH_2), 1.59 (2H, m, CH_2), 1.00 (3H, t, $^3J_{HH}$ 7.2 Hz). EtOAc (4.12 (q), 2.05 (s), 1.26 (t)) *n*-pentane (0.88 (t), 1.27 (m)), benzoic acid (9H, 8.18-8.07, 7.70-7.43). The product was obtained as a white powder (125 mg / 55%). NP HPLC retention times of enantiomers: 18.2 and 18.8 minutes.

2c 2-oxoheptane-1,3-diyl dibenzoate:
^{1}H NMR (400MHz; $CDCl_3$) δ 8.11-8.07 (4H, m, CH_{ortho}), 7.64-7.56 (2H, m, CH_{para}), 7.50-7.43 ((4H, m, CH_{meta}), 5.49 (1H, m, C*H*OR), 5.21 (1H, d, $^2J_{HH}$ 17.2 Hz, C*H*HOR), 5.06 ((1H, d, $^2J_{HH}$ 17.2 Hz, CH*H*OR), 1.54-1.47 (2H, m, CH_2), 1.43-1.38 (2H, m, CH_2), 1.32 -1.29 (2H, m, CH_2), 1.26 (3H, t, $^3H_{HH}$ 7.2 Hz). EtOAc (4.12 (q), 2.05 (s), 1.26 (t)) *n*-pentane (0.88 (t), 1.27 (m)). The product was obtained as a white powder (50 mg / 21%). NP HPLC retention times of enantiomers: 15.7 and 17.1 minutes.

3c 4-methyl-2-oxopentane-1,3-diyl dibenzoate:
^{1}H NMR (400MHz; $CDCl_3$) δ 8.10 (4H, m,$^3J_{HH}$ 8.4 Hz, $^4J_{HH}$ 1.2 Hz, CH_{ortho}), 7.61 (2H, m, CH_{para}), 7.46 (4H, m, $^3J_{HH}$ 8.4 Hz, CH_{meta}), 5.33 (1H, d, $^3J_{HH}$ 4.8 Hz, C*H*OR), 5.24 (1H, d, $^2J_{HH}$ 17.2 Hz, CH*H*OR), 5.05 (1H, d, $^2J_{HH}$ 17.2 Hz, C*H*HOR), 2.49 (1H, m, C$H(CH_3)_2$), 1.16 (3H, d, $^3J_{HH}$ 6.8 Hz, CH_3), 1.14 (3H, d, $^3J_{HH}$ 6.8 Hz, CH_3). EtOAc (4.12 (q), 2.05 (s), 1.26 (t)), benzoic acid (8.12, 7.7-7.6, 7.5-7.4).
The product was obtained as a pale yellow oil ($\approx$ 150 mg / 70%). NP HPLC retention times of enantiomers: 18.3 and 19.5 minutes.

4c 1-cycloheyl-2-oxopropane-1,3-diyl dibenzoate:
the product was not synthesised due to unsuccessful conversion of the substrate by the enzyme.

1.2.4 Glycolaldehyde Activity Assay

Cell free extract (50 µL) was incubated with the cofactors (ThDP: 1 mM, Mg^{2+}: 4 mM, 20 min., 800 rpm, 25°C). Subsequently lithium hydroxypyruvate and glycolaldehyde were added at a final concentration of 50 mM in 300 µL total reaction volume. The reaction mixture was shaken (15 min., 800 rpm, 25°C), quenched by 1:1 addition of trifluoroacetic acid (300 µL, 0.2% v/v), the enzyme removed by centrifugation and the supernatant was analysed by RP HPLC. Erythrulose product concentrations were obtained using a calibration curve ($R^2 = 0.998$) and subsequently were converted into moles. The activity was determined according to the definition of 1U = 1µmol/minute. The activity was multiplied by 20 to correct for 50 µL cell free extract in order to obtain the volumetric activity in $\frac{U}{mL}$[1].

1.2.5 Preparative Scale Bioconversions

Cell free extract (20 U based on the glycolaldehyde activity assay) was incubated with the transketolase cofactors (20 min, room temperature, final concentrations: Mg^{2+} 18 mM, ThDP 5 mM). Lithium hydroxypyruvate (110 mg, 1.0 mmol, 1.0 eq.) and the corresponding aldehyde (1.0 mmol, 1.0 eq.) were added and the reaction volume was adjusted to 10 mL by addition of buffer (5 mM sodium phosphate, pH = 7.0). The flask was sealed air tight and the reaction was carrried out overnight in a shaker (25°C, 200 rpm, $\approx$ 18h). The reaction mixture was transferred into a 50 mL falcon tube, extracted with MTBE (2x, 40 mL) and the solvent was removed *in vacuo*. The product was obtained in moderate to good purity for low activity mutants and excellent purity with D477E (determined by ^{1}H NMR).

1.2.6 Determination of Michaelis-Menten Parameters

Aqueous solutions of racemic dihydroxyketone products were prepared in duplicate and subsequently used in dilution series to obtain HPLC calibration curves. In order to measure the Michaelis-Menten parameters under credible *initial rate conditions* (<20% conversion), individual reaction times were determined for all enzymes and substrates by following the conversion over time. The buffered reaction mixture (600 µL, 5 mM sodium phosphate, pH = 7.0) containing holotransketolase (100 µg/674 pmol), lithium hydroxypyruvate (65 mM) and the corresponding aldehyde (30 mM) was incubated in a thermoshaker (500 rpm, 25°C). Samples were taken over time (50µL, quenched by 1:1 addition of 0.2% v/v TFA), the enzyme precipitated by centrifugation and the conversion was analysed by RP HPLC.

Substrate	WT	D477E	D477T	R528K	R528Q	R528K/ S527T	R528Q/ S527T
**1a**	6h	10 min.	6h	5h	3h	7h	5h
**2a**	6h	10 min.	6h	5h	3h	7h	5h
**3a**	21h	21h	21h	21h	21h	21h	21h
**4a**	50h	50h	50h	50h	50h	50h	50h

Table S 2: Individual reaction times for <20% conversion.

In order to determine the Michaelis-Menten parameters, buffered reaction mixtures (300 µL, 5 mM sodium phosphate, pH = 7.0) containing the holoenzyme (50 µg, 1 mM ThDP, 4 mM Mg^{2+}), lithium hydroxypyruvate (100 mM) and the corresponding aldehyde at different concentrations (5, 10, 20, 40, 70, 100, 150 mM) were incubated (25 °C, 500 rpm) *in duplicate* over the individual times previously determined. The reactions were quenched by 1:1 addition of 0.2% (v/v) TFA, the enzyme was precipitated by centrifugation and product concentrations were determined RP HPLC analysis. Product concentrations were correlated to molar amounts and converted into rates by taking the reaction time into account. The average from duplicate data was then plotted against substrate concentration. A Michaelis-Menten type non-linear fit was obtained by inserting literature based initial parameters for K_M and v_{max} into equation (3). The Excel built-in solver then automatically varied K_M and v_{max} in order to successively minimise the sum of the squared errors between the measured and fitted data points until the solver converged to the best solution for K_M and v_{max}. The parameter k_{cat} was obtained according to equation (4) taking the total enzyme concentration (50 µg / 337 pmol) into account.

$$\vartheta_0 = \frac{v_{max} \cdot [S]}{K_M + [S]} \tag{3}$$

$$k_{cat} = \frac{v_{max}}{[E]_0} \tag{4}$$

1.2.7 Equilibrium Analysis by NMR

A benzene-D_6 capillary was obtained commercially (Sigma-Aldrich) and was used as internal standard in NMR water suppression experiments using the PRESAT pulse sequence in sealed Wilmad screw-cap NMR tubes (Sigma Aldrich). The benzene signal (s, 7.16 ppm) was used as reference and its integral was normalised to 1000. The characteristic signals of the dihydroxyketone motif 4.61 (1H, d, $^2J_{HH}$ 19.6 Hz), 4.52 (1H, d, $^2J_{HH}$ 19.6 Hz) were used to follow the erythrulose concentration over time.

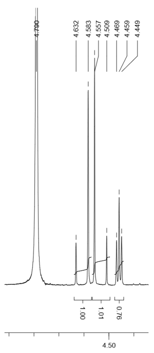

Figure S 3: Characteristic peaks of L-erythrulose.

Enzyme (WT transketolase, 200 µg) was incubated with its cofactors (25 °C, 500 rpm, 20 min, final concentrations: ThDP 5 mM, Mg^{2+} 18 mM).
a) Solutions of glycolaldehyde and lithium hydroxypyruvate were added to achieve a final concentration of 100 mM each and the reaction mixture was filled up to 500 µL with buffer (5 mM sodium phosphate, pH = 7.0).
b) A solution of glycolaldehyde was added to achieve a final concentration of 200 mM and the reaction mixture was filled up to 500 µL with buffer (5 mM sodium phosphate, pH = 7.0).
c) Three control reactions were prepared in buffered solutions containing the cofactors Mg^{2+} and ThDP containing 1) glycolaldehyde (200 mM), 2) glycolaldehyde (100 mM) with LiHPA (100 mM) and 3) L(+)erythrulose (100 mM).

1.3 Modelling and *in silico* Methods

1.3.1 Thermodynamic Model

A thermodynamic model was created using MATLAB (MathWorks, Version R2014b) to describe the equilibrium concentrations of glycolaldehyde in aqueous solution as shown in figure (4) based on experimental data obtained from NMR studies by Kua et al.[5]. Experimental equilibrium concentrations were used to assign arbitrary kinetic parameters according to the *Law of mass action* as shown in equation (6) for each equilibrium.

$$aA + bB \underset{k2}{\overset{k1}{\rightleftarrows}} cC \tag{5}$$

$$K_{eq} = \frac{[C]^c}{[A]^a \cdot [B]^b} = \frac{k1}{k2} \tag{6}$$

The change in Gibbs free energy for the one-substrate transketolase reaction was calculated according to equation (7) using the Gibbs free energies of formation of the substrates and products shown in table (3). The formation of erythrulose from LiHPA via decarboxylation was implemented as irreversible in the model. The free energy change of the one-substrate reaction was then correlated to the corresponding equilibrium ratio using equation (9).

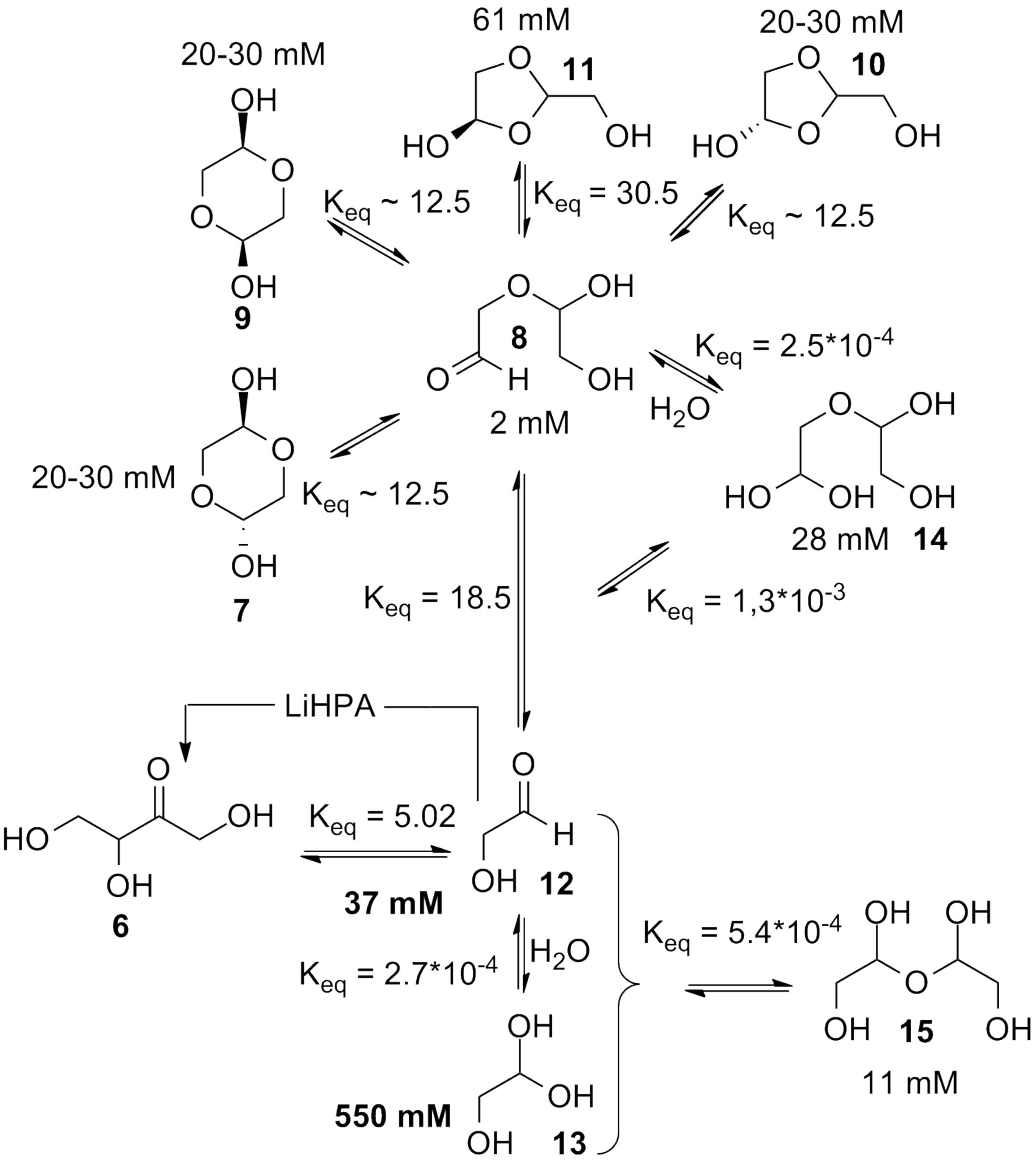

Figure S 4: Equilibrium relationships of the dominant species of glycolaldehyde in aqueous solution coupled to the one-substrate transketolase reaction of glycolaldehyde based on data published by Kua et al.[5].

$$\Delta_r G^0 = \sum \Delta G_f^0(products) - \sum \Delta G_f^0(substrates) \quad (7)$$

	glycolald.	HPA	H_2O	erythrulose	HCO_3^-
ΔG_f^0 $[\frac{kJ}{mol}]$	-29,2[6]	-118,4[7]	-237,1[8]	-62,4[9]	-586,9[8]

Table S 3: Gibbs Free Energies of substrates and products involved in the transketolase mediated synthesis of erythrulose.

The system was then described as a coupled set of first order (10) and second order (11) differential equations, allowing the system to converge towards thermodynamic equilibrium from any starting point of choice.

$$\Delta_r G^0 = -RT \ln K_{eq} \quad (8)$$

$$K_{eq} = \exp(\frac{-\Delta_r G^0}{RT}) \quad (9)$$

$$\frac{d[A]}{dt} = -k \cdot [A] \quad (10)$$

$$\frac{d[A]}{dt} = -k \cdot [A] \cdot [B] \quad (11)$$

1.3.2 Computational Docking Studies with YASARA

In silico docking studies were carried out with the programme YASARA[10] (Version 16.2.18) using the crystal structures 1TRK and 1GPU for *S. cerevisiae* transketolase and 1QGD for *E. coli* transketolase. The simulation box was defined at a size of 10 Å around the thiamin C2 in 1TRK and the ylid anion in 1GPU. Three dimensional structure files of the substrates were energy minimised with ChemBio3D Ultra 12.0 (Cambridgesoft) using MM2 energy minimisation and saved in the .pdb file format. Mutant crystal structures were made with PyMOL chosing the residue's most probable rotamer. In the case of the mutant D477E, the resulting crystal structure was subsequently energy minimised using YASARA. Structural alignment of crystal structures 1QGD (*E. coli*) and 1TRK (*S. cerevisiae*) and calculation of the RMSD was performed in PyMOL. Protein images were created using PyMOL.

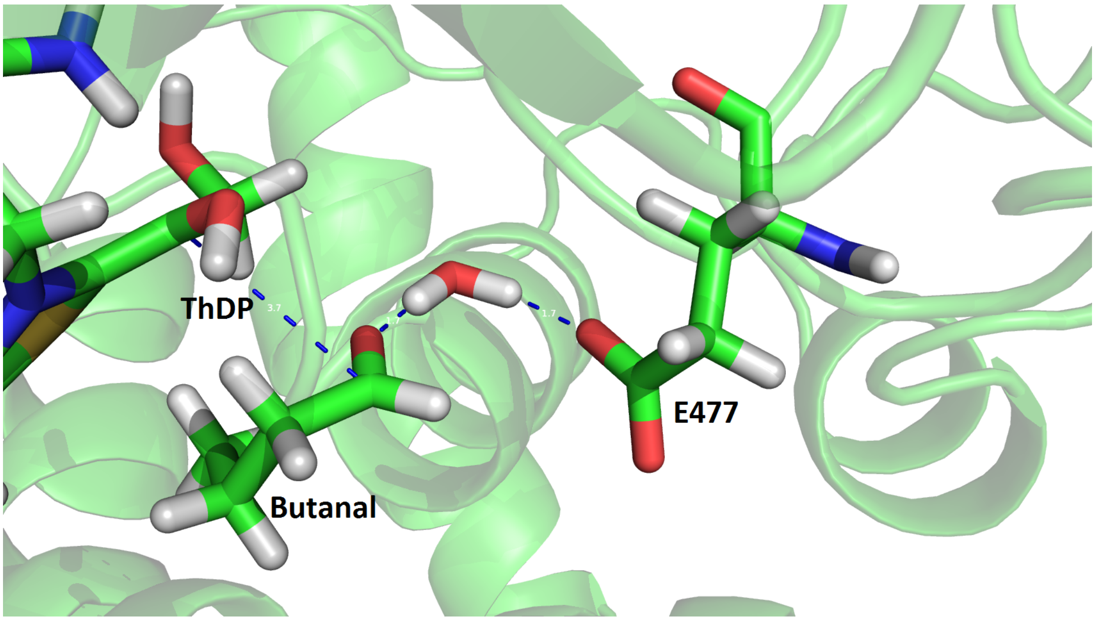

Figure S 5: Butanal docked into the energy minimised model containing mutation D477E.

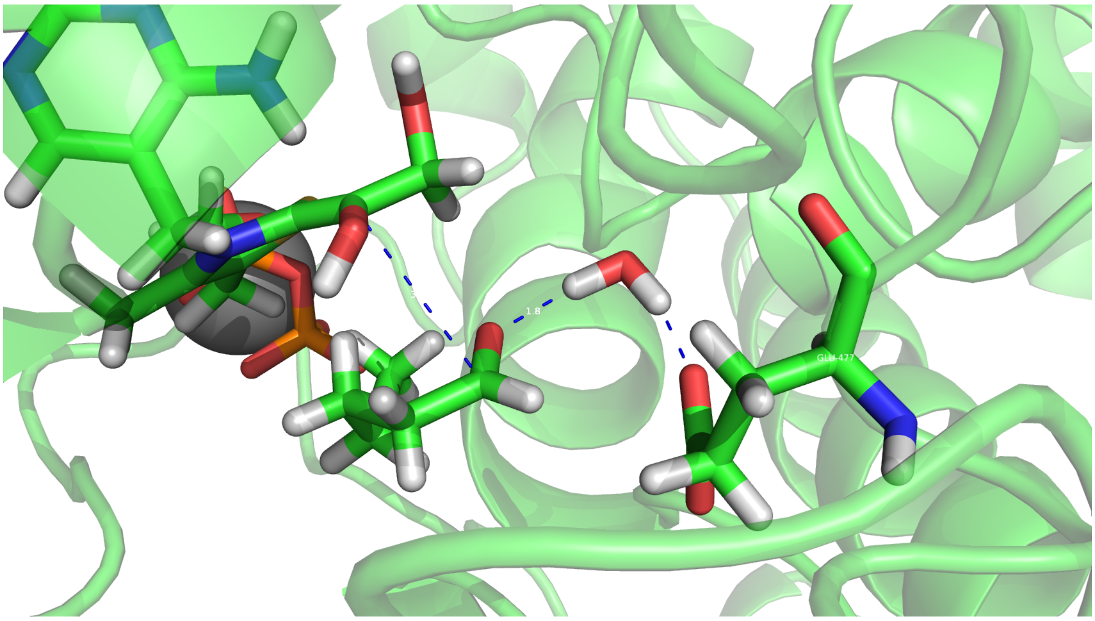

Figure S 6: Pentanal docked into the energy minimised model containing mutation D477E.

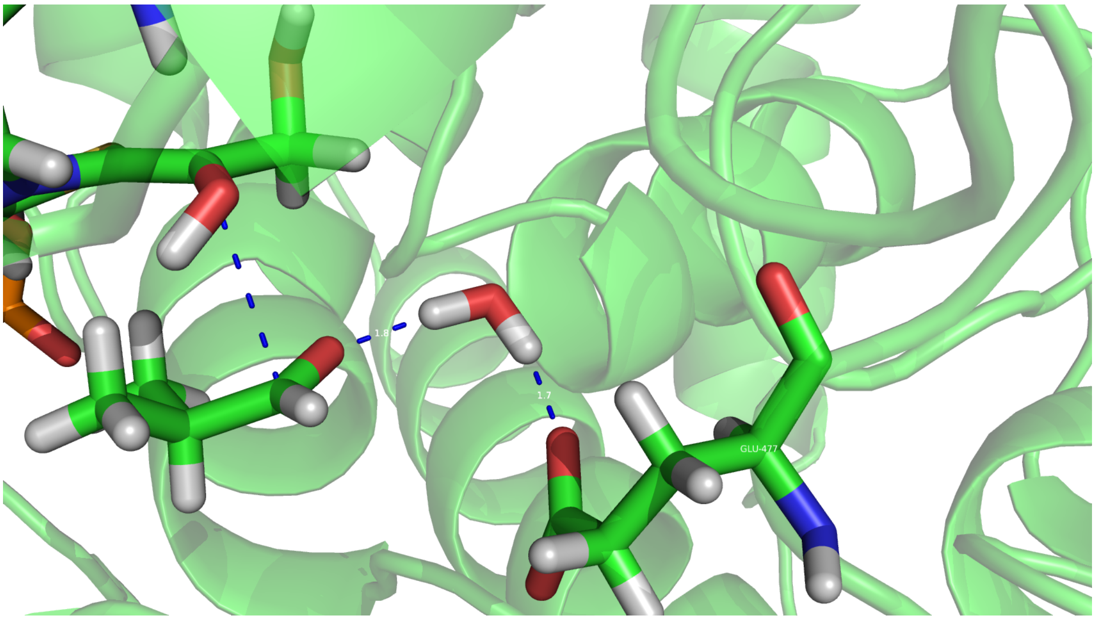

Figure S 7: Isobutanal docked into the energy minimised model containing mutation D477E.

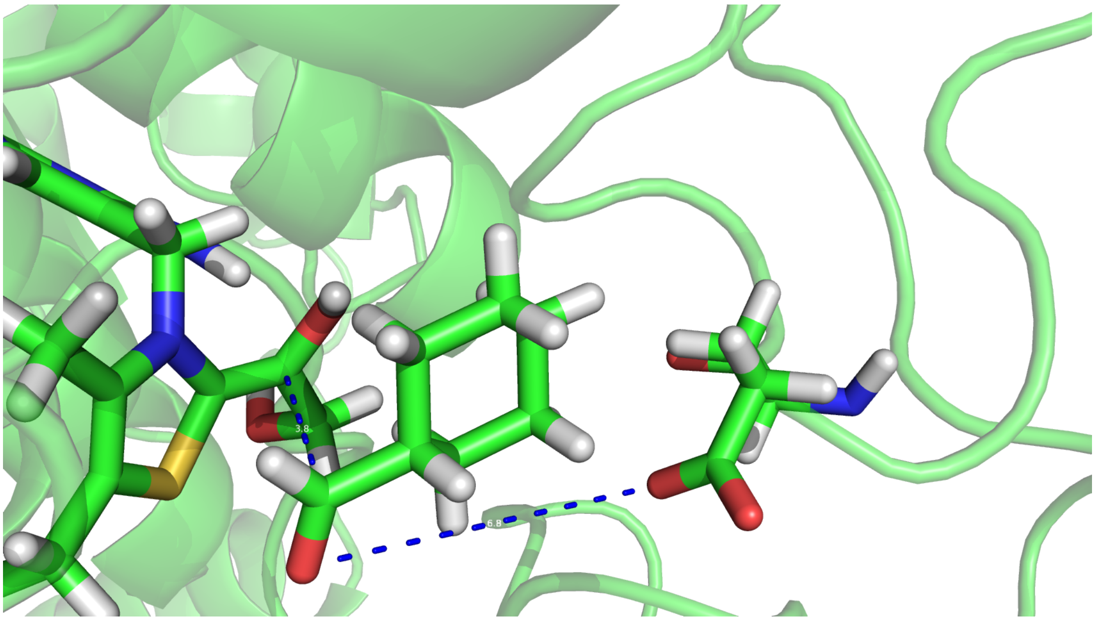

Figure S 8: Cyclohexane carboxaldehyde docked into the energy minimised model containing mutation D477E. No interactions via a bridging molecule of water are predicted.

2 References

1 A. Ranoux, S. K. Karmee, J. Jin, A. Bhaduri, A. Caiazzo, I. W. C. E. Arends and U. Hanefeld, *ChemBioChem*, 2012, **13**, 1921-1931.

2 K. G. Morris, M. E. B. Smith and N. J. Turner, *Tetrahedron: Asymmetry*, 1996, **7**, 2185-2188.

3 A. Cazares, J. L. Galman, L. G. Crago, M. E. B. Smith, J. Strafford, L. Rios-Solis, G. J. Lye, P. A. Dalby and H. C. Hailes, *Org. Biomol. Chem.*, 2010, **8**, 1301-1309.

4 C. Richter, F. Berndt, T. Kunde and R. Mahrwald, *Org. Lett.*, 2016, **18**, 2950-2953.

5 J. Kua, M. M. Galloway, K. D. Millage, J. E. Avila and D. O. De Haan, *J. Phys. Chem. A*, 2013, **117**, 2997-3008.

6 *Webpage*, 2016 http://biocyc.org/META/NEW-IMAGE?type=COMPOUND & object=GLYCOLALDEHYDE, (accessed April 25 2016).

7 *Webpage*, 2016 http://biocyc.org/META/new-image?object=OH-PYR, (accessed April 25 2016).

8 J. Speight in *Lange's Handbook of Chemistry*, McGraw-Hill Education, 2005.

9 *Webpage*, 2016 http://biocyc.org/META/NEW-IMAGE?type=COMPOUND & object=ERYTHRULOSE, (accessed April 25 2016).

10 E. Krieger and G. Vriend, *Bioinformatics*, 2014, **30**, 2981-2982.

3 Appendix

3.1 Protein purification

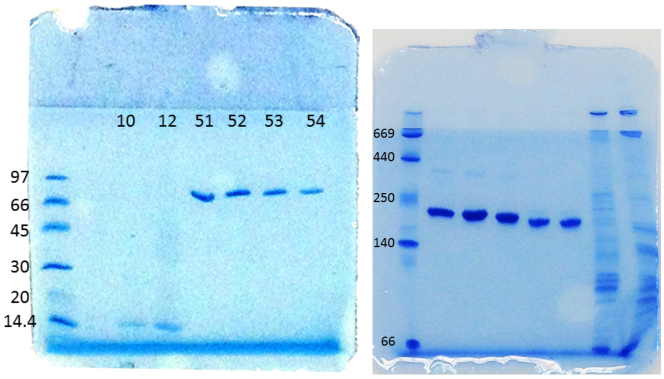

Figure S 9: SDS PAGE (left) and Native PAGE (right) of purified transketolase.

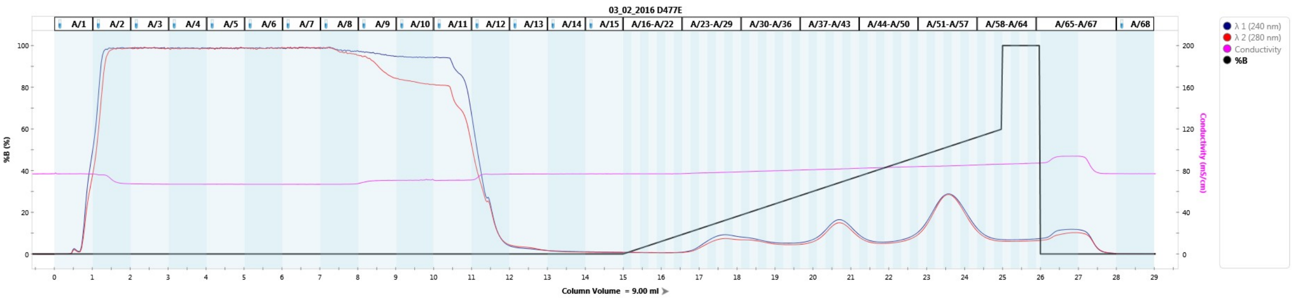

Figure S 10: Ni-NTA purification of D477E TK representative for WT and D477T TK.

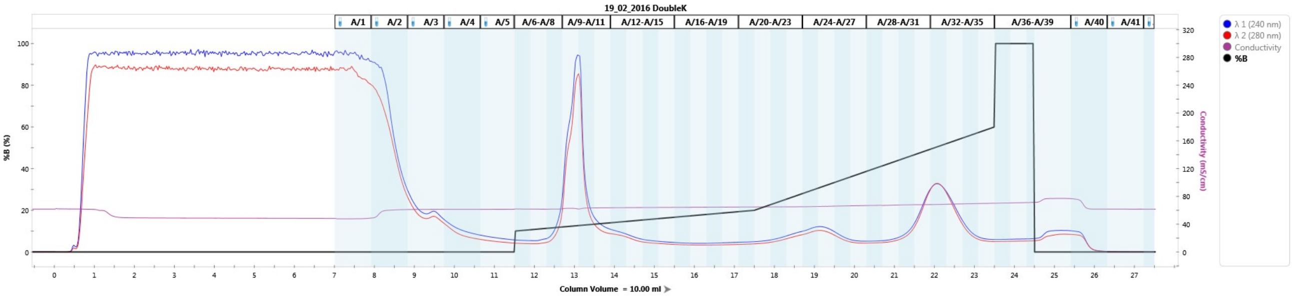

Figure S 11: Ni-NTA purification of R528K/S527T TK representative for R528K, R528Q and R528Q/S527T TK.

3.2 Chiral Analysis by HPLC

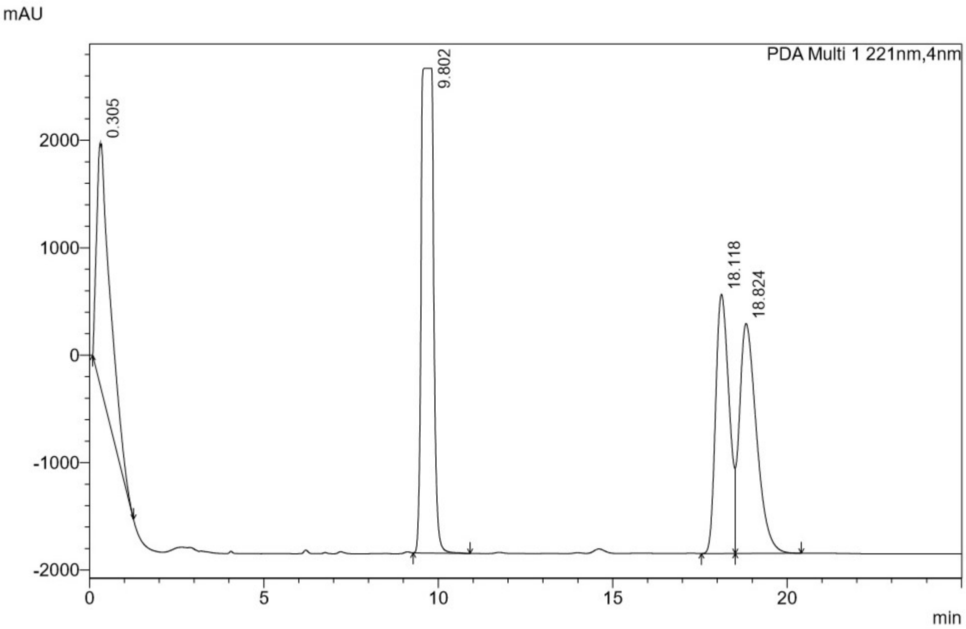

Figure S 12: Racemic standard of compound **1c** separated by chiral HPLC.

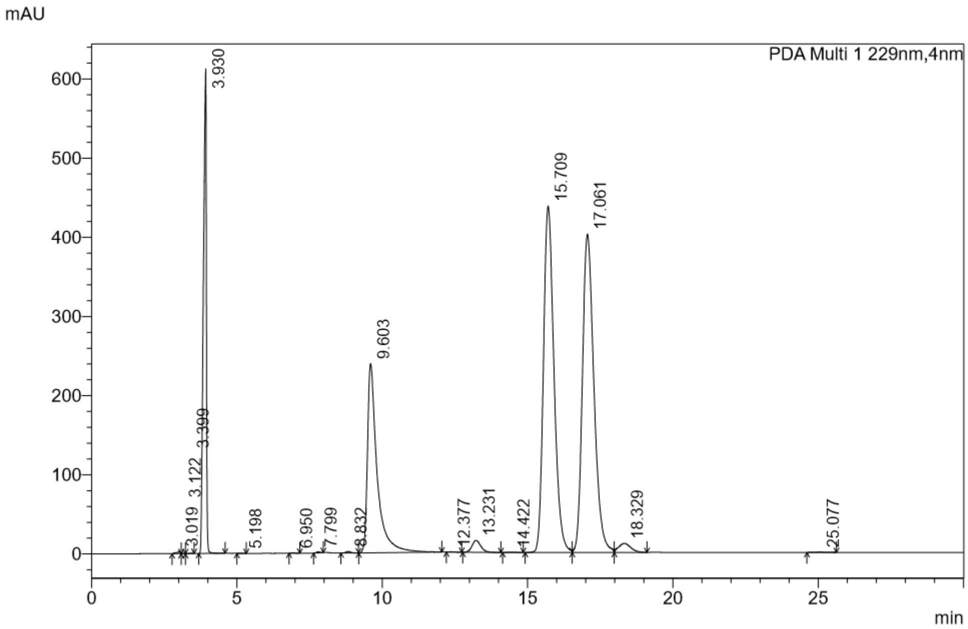

Figure S 13: Racemic standard of compound **2c** separated by chiral HPLC.

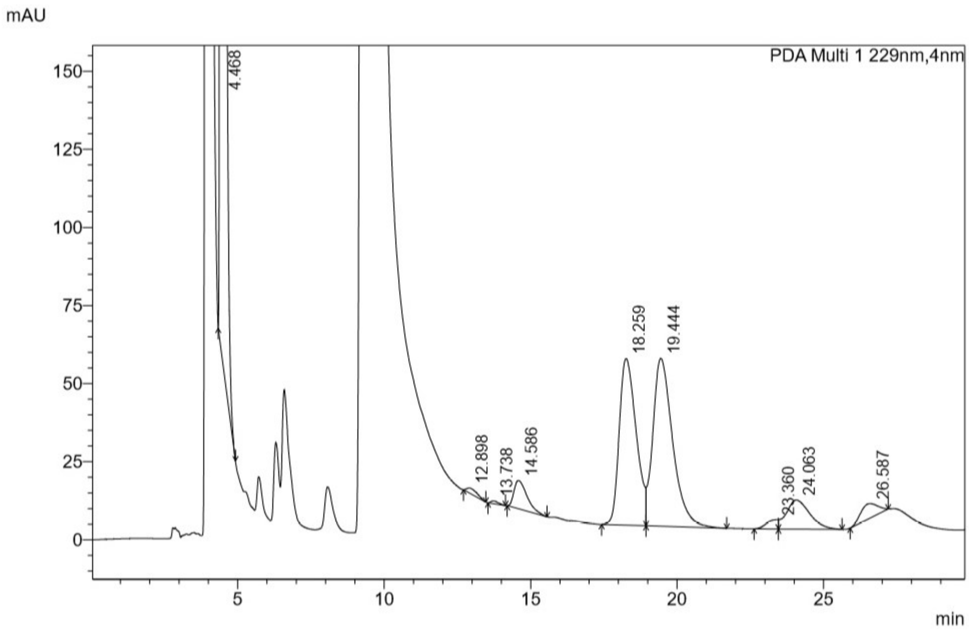

Figure S 14: Racemic standard of compound **3c** separated by chiral HPLC.

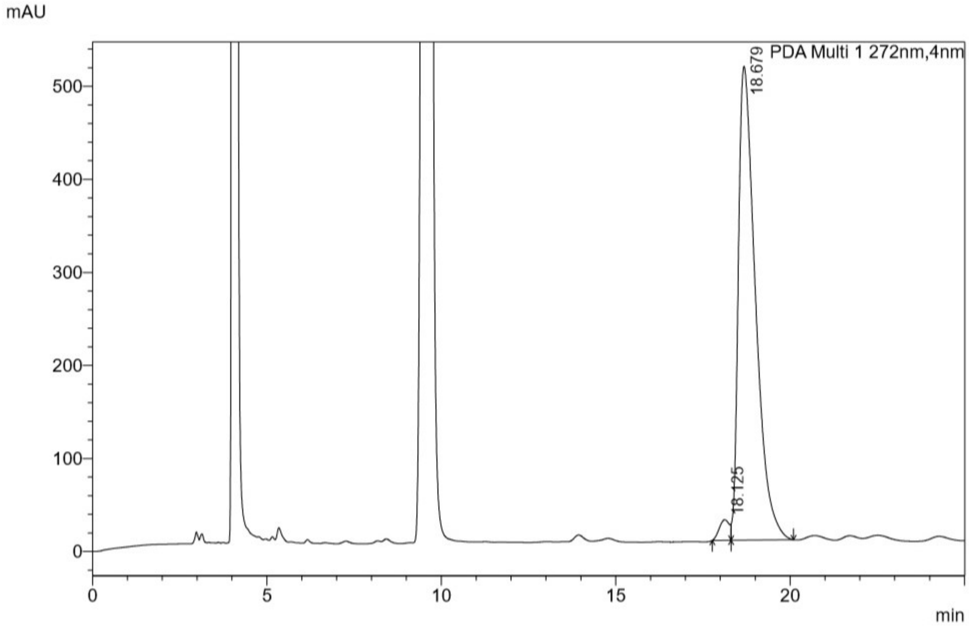

Figure S 15: Determination of the *ee* for compound **1c** by chiral HPLC.

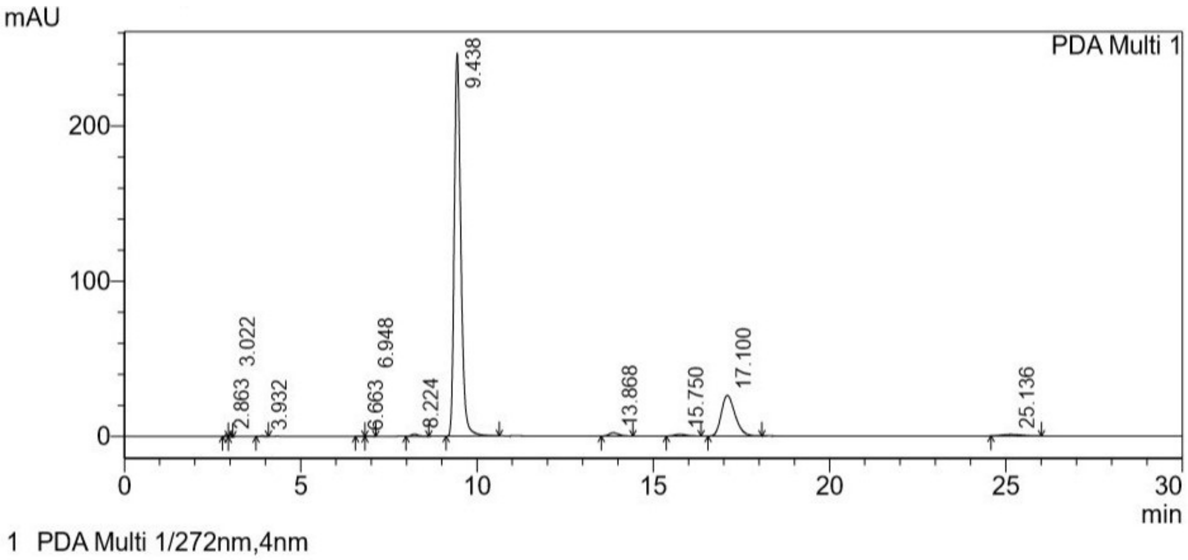

Figure S 16: Determination of the *ee* for compound **2c** by chiral HPLC.

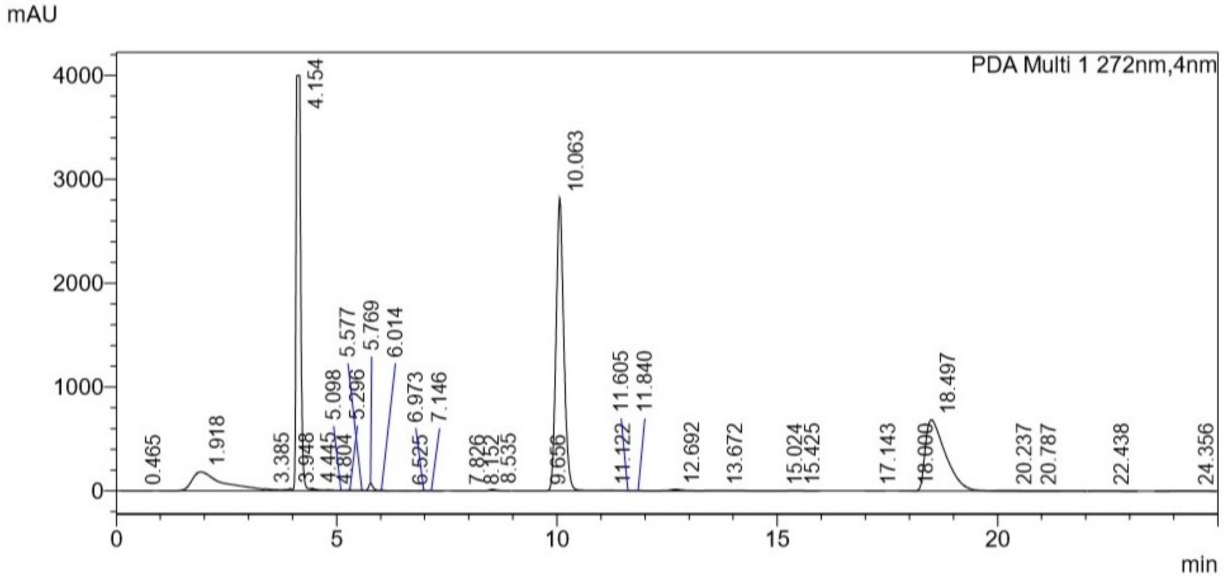

Figure S 17: Determination of the *ee* for compound **3c** by chiral HPLC.

3.3 Gene and Primer Sequences

**XXX**:	WT (GAT)	D477E (GAA)	D477T (ACT)
**YYY**:	WT (AGC)	S527T (ACC)	
**ZZZ**:	WT (CGT)	R528K (AAG)	R528Q (CAG)

Table S 4: Overview of point mutation sequences.

Codon optimised gene sequence of *S.cerevisiae* transketolase (gene accession number P23254) with indicated **mutations** according to table (4).

ATGACCCAGTTTACCGATATTGATAAACTGGCAGTTAGCACCATT
CGTATTCTGGCAGTTGATACCGTTAGCAAAGCAAATAGTGGTCAT
CCGGGTGCACCGCTGGGTATGGCACCGGCAGCACATGTTCTGTGG
TCACAGATGCGTATGAATCCGACAAATCCGGATTGGATTAATCGT
GATCGTTTTGTTCTGAGCAATGGTCATGCAGTTGCACTGCTGTAT
AGCATGCTGCATCTGACCGGTTATGATCTGAGCATTGAAGATCTG
AAACAGTTTCGTCAGCTGGGTAGCCGTACACCGGGTCATCCGGAA
TTTGAACTGCCTGGTGTTGAAGTTACCACCGGTCCGCTGGGTCAG
GGTATTAGCAATGCAGTTGGTATGGCAATGGCACAGGCAAATCTG
GCAGCAACCTATAATAAACCGGGTTTTACCCTGAGCGATAATTAT
ACCTATGTGTTTCTGGGCGATGGTTGTCTGCAGGAAGGTATTAGC
AGCGAAGCAAGCAGCCTGGCAGGTCATCTGAAACTGGGTAATCTG
ATTGCCATTTATGATGATAATAAAATTACCATTGATGGTGCGACC
AGCATTAGCTTTGATGAAGATGTTGCCAAACGCTATGAAGCATAT
GGTTGGGAAGTTCTGTATGTGGAAAATGGTAATGAAGATCTGGCA
GGTATTGCAAAAGCAATTGCACAGGCCAAACTGAGCAAAGATAAA
CCGACCCTGATTAAAATGACCACCACCATTGGTTATGGTAGCCTG
CATGCTGGTAGCCATAGCGTTCATGGCGCACCGCTGAAAGCAGAT
GATGTTAAACAGCTGAAAAGCAAATTTGGCTTTAACCCGGATAAA
AGCTTTGTTGTTCCGCAGGAAGTGTACGATCATTATCAGAAAACC
ATTCTGAAACCGGGTGTGGAAGCCAATAATAAATGGAACAAACTG
TTTAGCGAATATCAGAAAAAATTTCCGGAACTGGGTGCAGAACTG
GCACGTCGTCTGAGCGGTCAGCTGCCGGCAAATTGGGAAAGCAAA
CTGCCGACCTATACCGCAAAAGATAGCGCAGTTGCAACCCGTAAA
CTGAGCGAAACCGTTCTGGAAGATGTTTATAATCAGCTGCCGGAA
CTGATTGGTGGTAGCGCAGATCTGACCCCGAGCAATCTGACCCGT
TGGAAAGAAGCACTGGATTTTCAGCCGCCGAGCAGCGGTAGCGGT
AATTATTCAGGTCGTTATATTCGCTATGGCATTCGTGAACATGCA
ATGGGTGCAATTATGAATGGTATTAGCGCATTTGGTGCCAATTAT
AAACCGTATGGTGGCACCTTTCTGAATTTTGTTAGCTATGCAGCC

GGTGCAGTTCGTCTGAGCGCACTGAGTGGTCATCCGGTTATTTGG
GTTGCGACCCATGATAGCATTGGTGTTGGTGAA**XXX**GGTCCGACC
CATCAGCCGATTGAAACCCTGGCACATTTTCGTAGCCTGCCGAAT
ATTCAGGTTTGGCGTCCGGCAGATGGCAATGAAGTTAGCGCAGCA
TATAAAAATAGCCTGGAAAGCAAACATACCCCGAGCATTATTGCA
CTG**YYYZZZ**CAGAATCTGCCGCAGCTGGAAGGTAGCAGCATTGAA
AGCGCAAGCAAAGGTGGTTATGTTCTGCAGGATGTTGCAAATCCG
GATATTATTCTGGTTGCAACCGGTAGCGAAGTTAGCCTGAGCGTT
GAAGCAGCAAAAACCCTGGCAGCCAAAAATATTAAAGCCCGTGTT
GTTTCACTGCCGGATTTTTTTACCTTTGATAAACAGCCGCTGGAA
TATCGTCTGAGCGTTCTGCCGGATAATGTTCCGATTATGAGCGTG
GAAGTTCTGGCAACCACCTGTTGGGGTAAATATGCACATCAGAGC
TTTGGTATTGATCGTTTTGGTGCAAGCGGTAAAGCACCGGAAGTG
TTTAAATTTTTTGGCTTTACACCGGAAGGTGTTGCAGAACGTGCA
CAGAAAACAATTGCCTTTTATAAAGGCGATAAACTGATTAGTCCG
CTGAAAAAAGCCTTTTAA

Primer sequence used for the site-directed mutagenesis D477E:

Forward primer:
CCCATGATAGCATTGGTGTTGGTGAAGAAGGTCCGACCCATCAGCCG

Reverse primer:
CGGCTGATGGGTCGGACCTTCTTCACCAACACCAATGCTATCATGGG

3.4 Michaelis-Menten Curve Fits

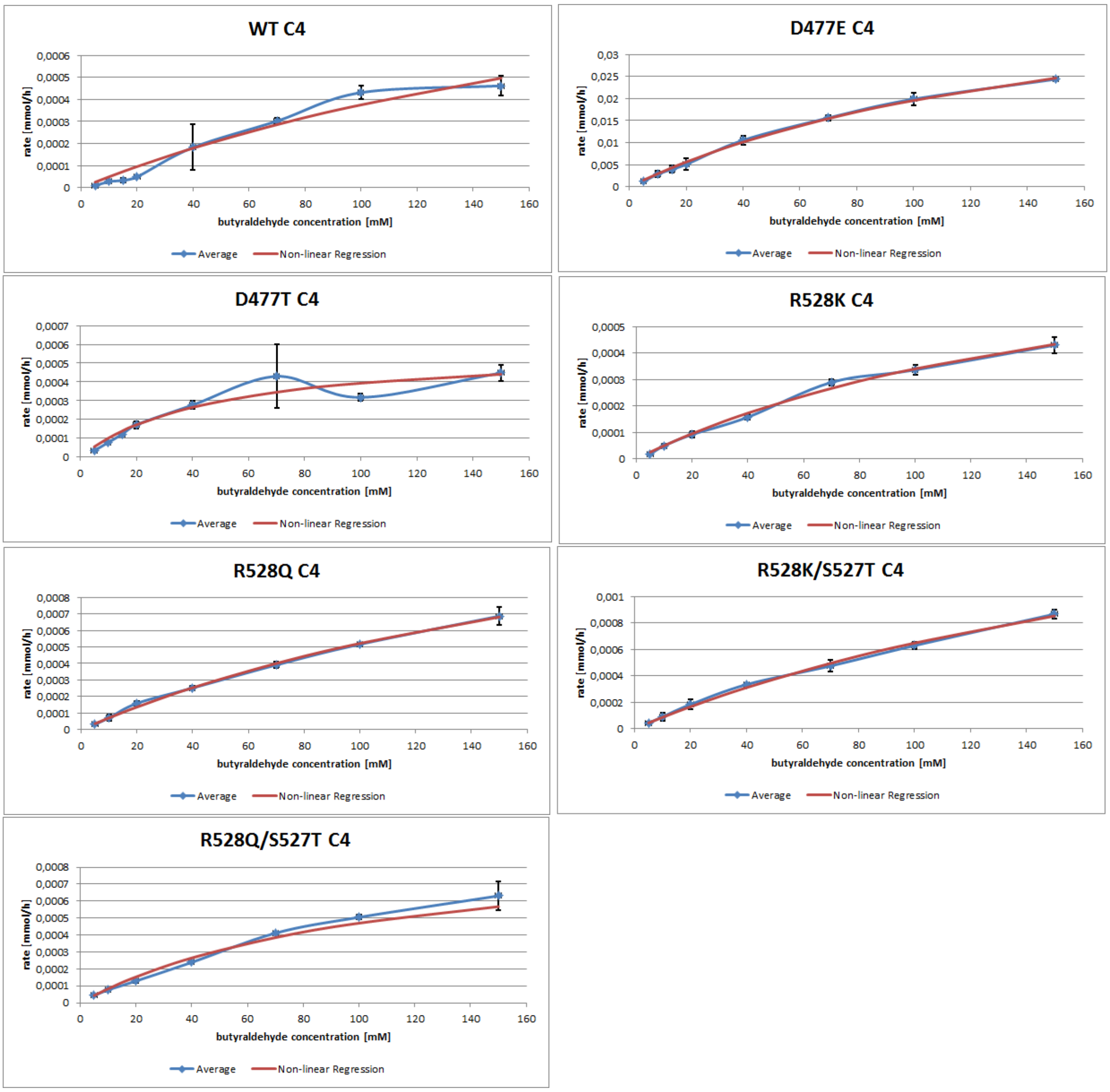

Figure S 18: Michaelis-Menten curve fits for product **1b**.

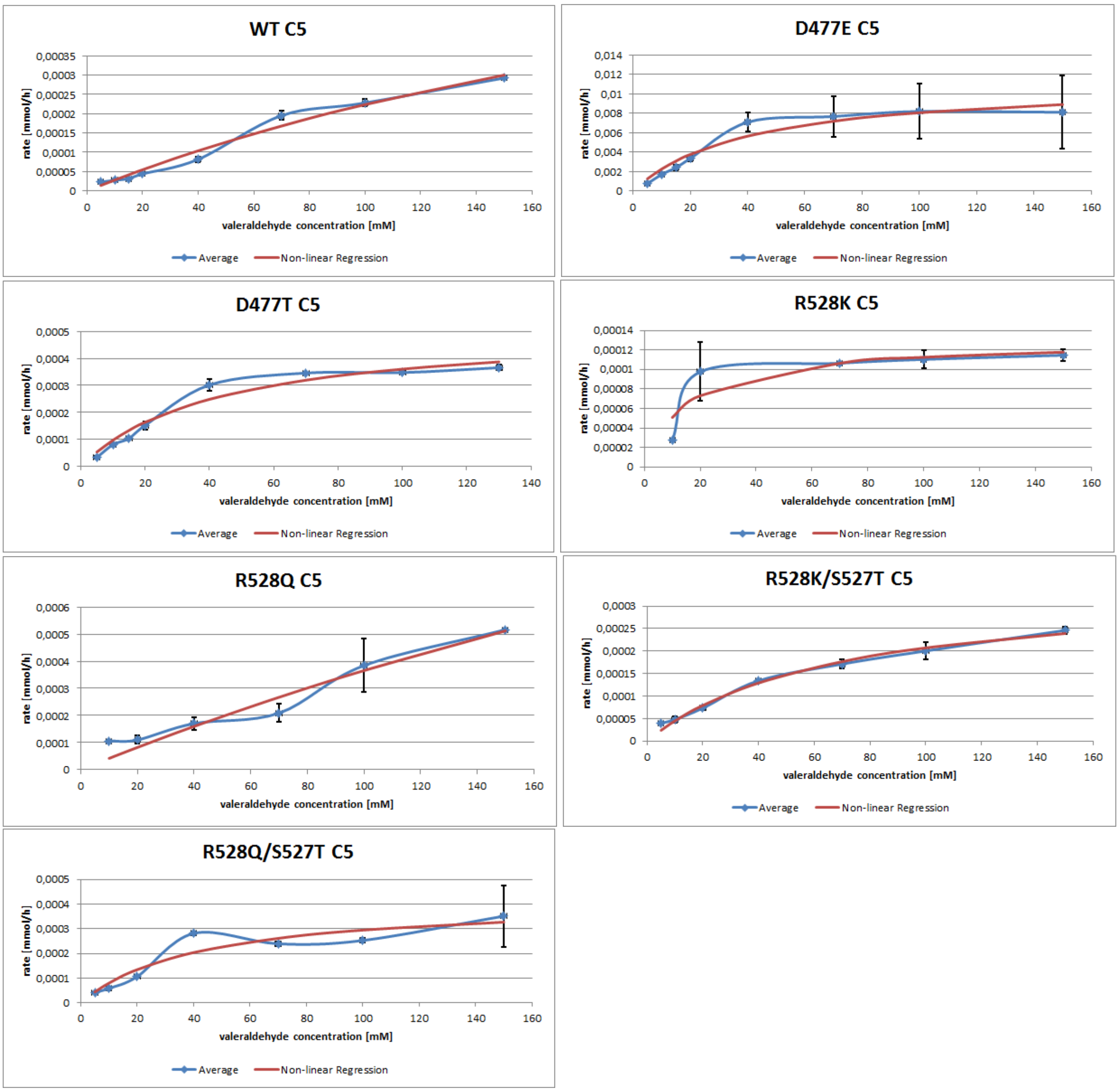

Figure S 19: Michaelis-Menten curve fits for product **2b**.

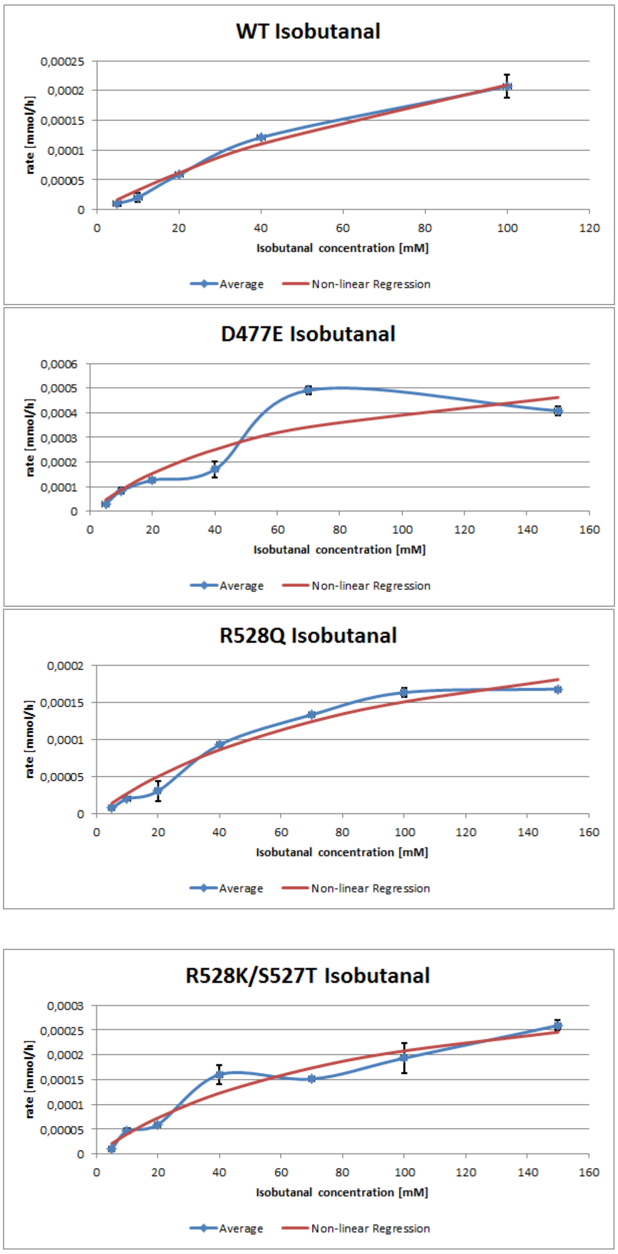

Figure S 20: Michaelis-Menten curve fits for product **3b**.

3.5 Thermodynamic Model Source Code

```
function Erythrulose_Synthesis_Glycolaldehyde_Equilibria

%this code is based on research published By Kua et al. "Glycolaldehyde
%monomer and oligomer in aqueous solution: Comparing computational
%chemistry and NMR data" The Journal of Physical Chemistry A, 117(14):
%2997-3008, 2013.

t = (0:0.001:6); %time vector [h]
y0 = [25; 0; 25; 0; 0; 0; 0; 0; 0; 55494; 100; 0];
% 1 2 3 4 5 Glycolaldehyde Hydrate 8 9 water LiHPA Erythrulose initial
%concentrations

opt = odeset('AbsTol',1e-10);
[t y]=ode45(@dydt,t,y0,opt);

plot(t,y(:,1),'r',t,y(:,2),'g',t,y(:,3),'r',t,y(:,4),'k',t,y(:,5),'y',...
    t,y(:,6),'-.',t,y(:,7),'--',t,y(:,8),'m',t,y(:,9),'c',t,y(:,11),'-',...
    t,y(:,12),'b')

legend('1','2','3','4','5','Glycolaldehyde','Glycolaldehyde Hydrate',...
    '8','9','LiHPA','Erythrulose')
xlabel('time [arbitrary]')
ylabel('concentration [mM]')

total = y(end,1)*2+y(end,2)*2+y(end,3)*2+y(end,4)*2+y(end,5)*2 ...
+y(end,6)+y(end,7)+y(end,8)*2+y(end,9)*2+y(end,12)*2;

disp(['c(Glycolaldehyde) = ' num2str(y(end,6)) ' mM at equilibrium'])
disp(['c(Glycolaldehyde_hydrate) = ' num2str(y(end,7))...
    ' mM at equilibrium'])
disp(['c(Erythrulose) = ' num2str(y(end,12)) ' mM at equilibrium'])

end

function dy = dydt(t,y)
k1 = 2;
k2 = 25;
k3 = 2;
k4 = 25;
k5 = 25;
k6 = 2;
k7 = 60;
k8 = 2;
k9 = 1.369; %37*37
k10 = 0.002;
k11 = 0.0550;
k12 = 205.3278; %37*55494 c(H2O) in water
k13 = 0.011;
k14 = 20.350; %37*550
k15 = 0.027;
k16 = 20.350; %37*550
k17 = 0.027;
```

```
k18 = 110.988; %2*55494
k19 = 5.02073;
k20 = 1;
k21 = 5; %Kinetic parameter for LiHPA involved synthesis (guessed)


dy(1) = -k1*y(1) + k2*y(2);
dy(2) =  k1*y(1) - k2*y(2) + k3*y(3) - k4*y(2) - k5*y(2) + k6*y(4) ...
 - k7*y(2) + k8*y(5) - k17*y(2)*y(10) + k18*y(8) -k9*y(2) + k10*y(6)*y(6);

dy(3) = -k3*y(3) + k4*y(2);
dy(4) = -k6*y(4) + k5*y(2);
dy(5) = -k8*y(5) + k7*y(2);
dy(6) = -2*k10*y(6)*y(6) + 2*k9*y(2) - k11*y(6)*y(10) + k12*y(7) ...
- k13*y(6)*y(7) + k14*y(9) - k15*y(6)*y(7) + k16*y(8) -k21*y(6)*y(11)...
-2*k19*y(6)*y(6) + 2*k20*y(12);

dy(7) =  k11*y(6)*y(10) - k12*y(7) - k13*y(6)*y(7) + k14*y(9)...
    - k15*y(6)*y(7) + k16*y(8);

dy(8) =  k17*y(2)*y(10) - k18*y(8) + k15*y(6)*y(7) -k16*y(8);
dy(9) =  k13*y(6)*y(7) - k14*y(9);
dy(10) = -k17*y(2)*y(10) +k18*y(8) -k11*y(6)*y(10) +k12*y(7);
dy(11) = -k21*y(6)*y(11);
dy(12) = k21*y(6)*y(11) + k19*y(6)*y(6) - k20*y(12);

dy = dy';
end
```

```
c(Glycolaldehyde) = 3.7023 mM at equilibrium
c(Glycolaldehyde_hydrate) = 55.0127 mM at equilibrium
c(Erythrulose) = 68.8606 mM at equilibrium
```

Published with MATLAB® R2014b

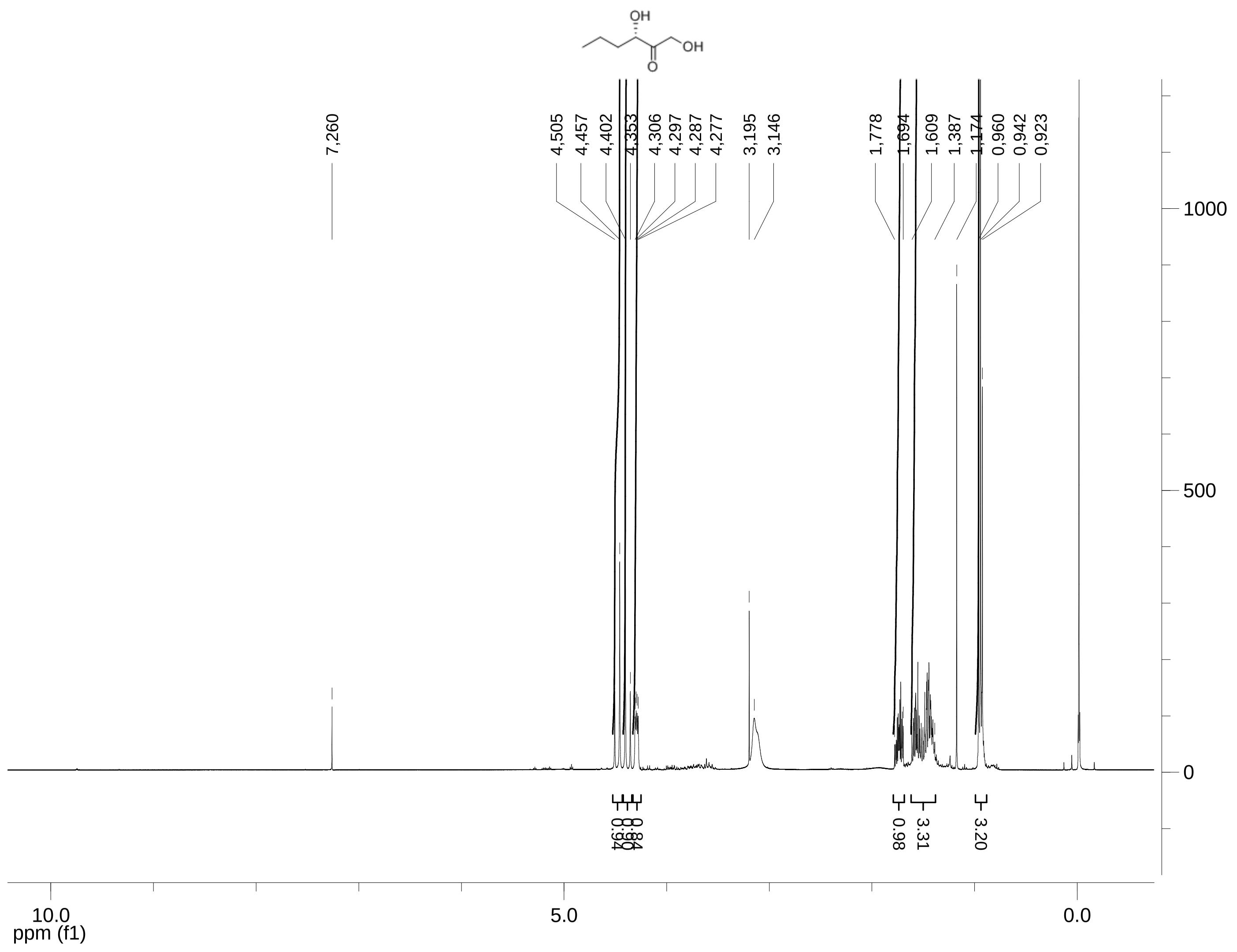

Figure S 21: **1b** 1,3-dihydroxyhexan-2-one produced enzymatically.

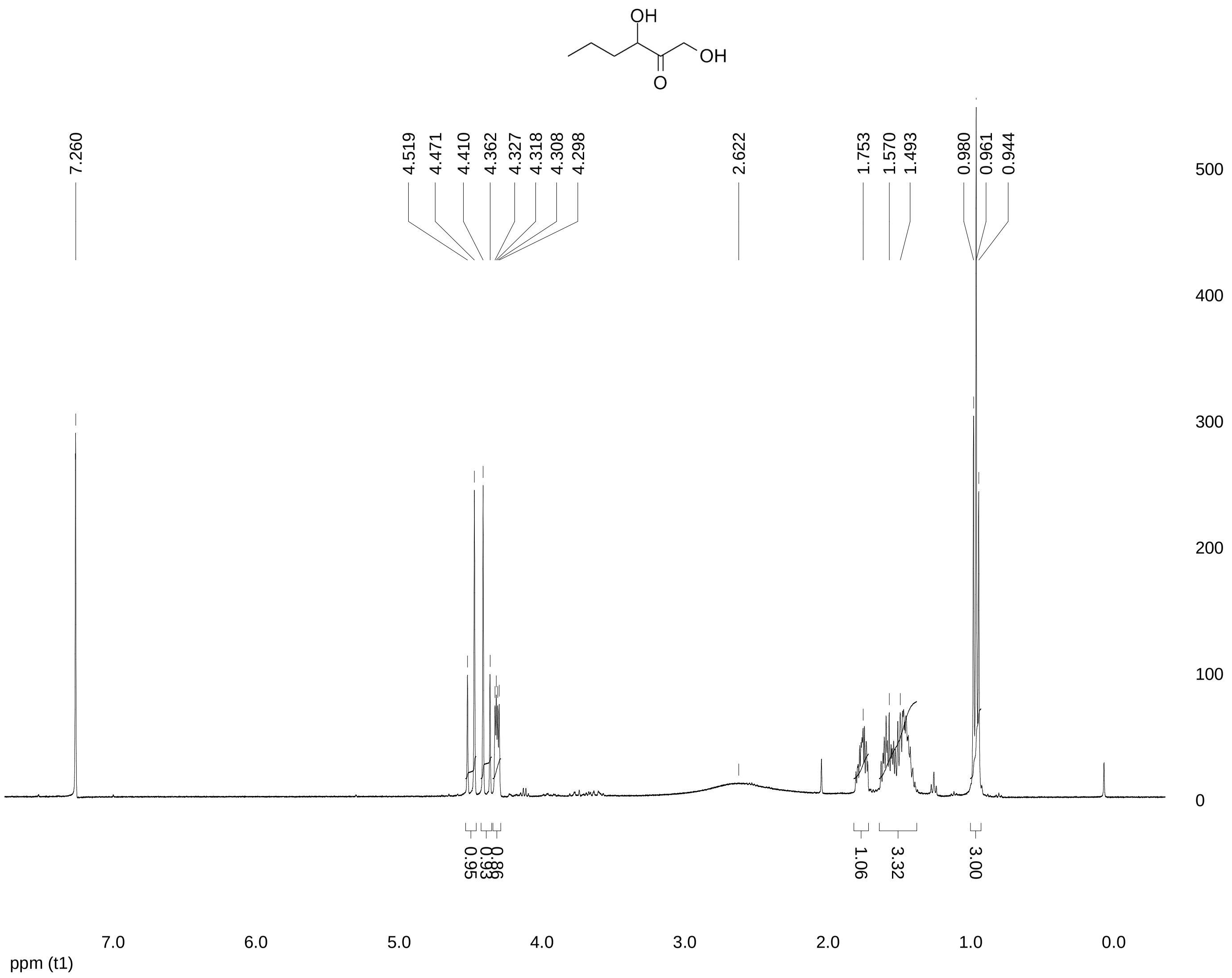

Figure S 22: Racemic **1b** 1,3-dihydroxyhexan-2-one produced by organocatalysis.

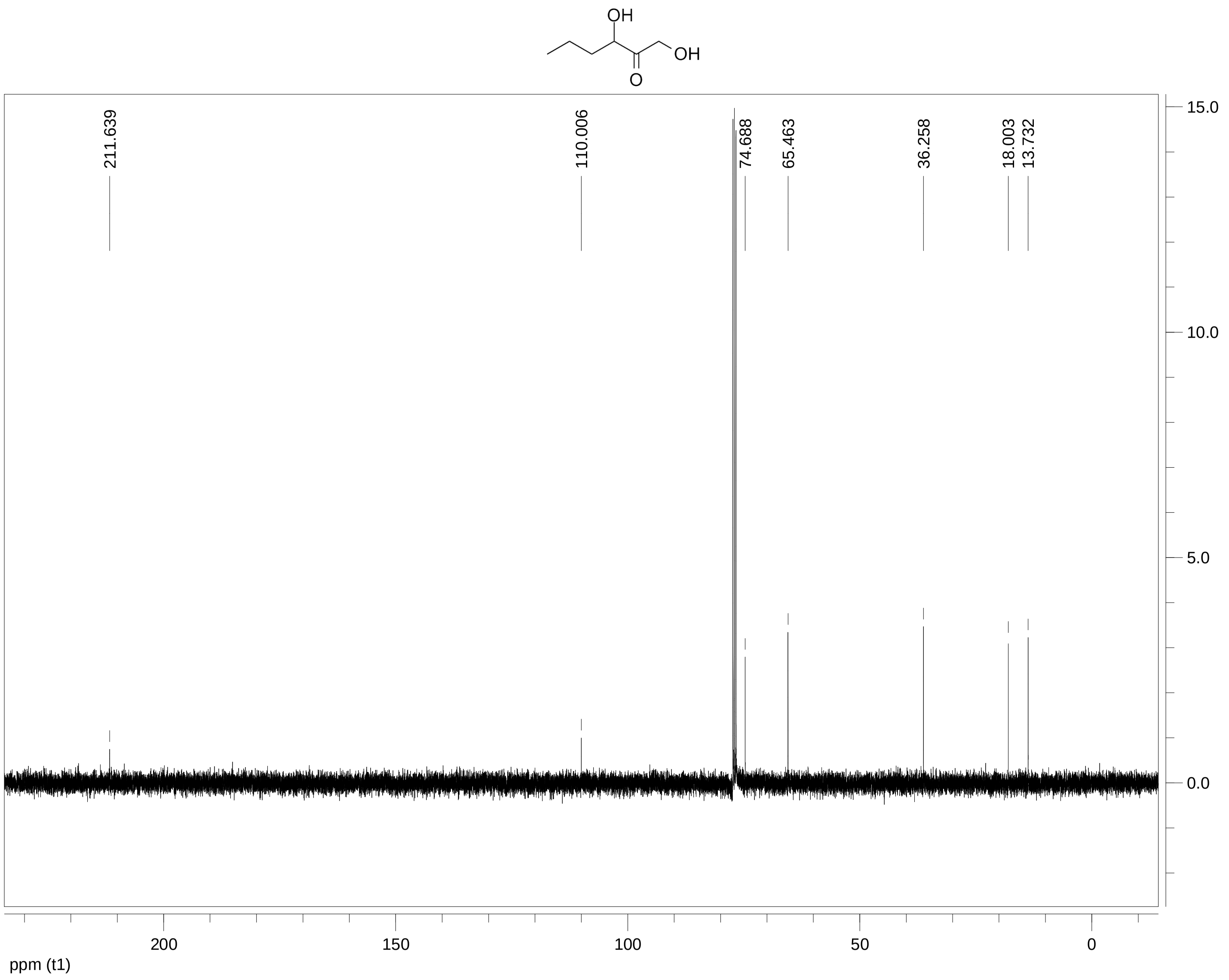

Figure S 23: Racemic **1b** 1,3-dihydroxyhexan-2-one. The peak at 110 ppm disappeared in a subsequent HMBC experiment.

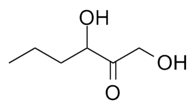

SM010_flash

Sample Name SM010_flash	Pulse sequence gHMBCAD	Temperature 25	Study owner BOC-Marsden
Date collected 2015-11-20	Solvent cdcl3	Spectrometer TUD-NMR400as-vnmrs400	Operator BOC-Marsden

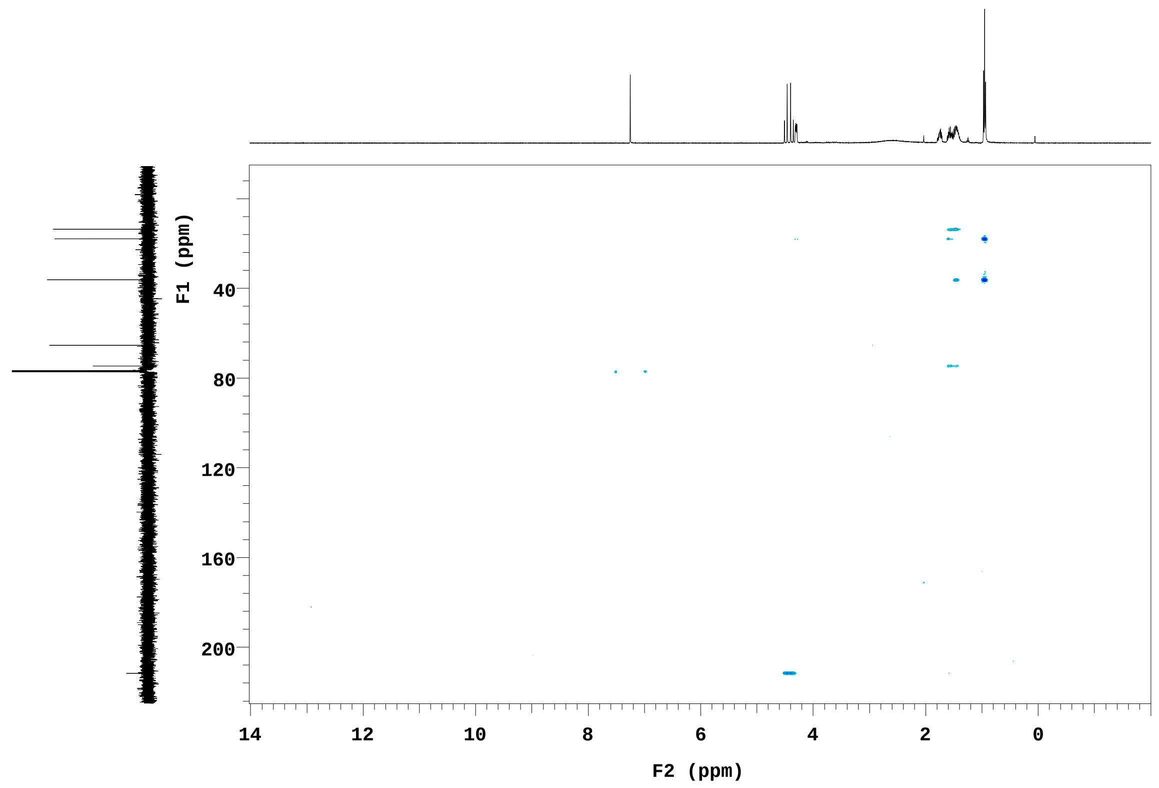

Data file /home/walk-in/vnmrsys/data/BOC-Marsden/SM010_flash01/SM010_flash_gHMBCAD_01.fid

Plot date 2015-11-24

Figure S 24: Racemic **1b** 1,3-dihydroxyheptan-2-one

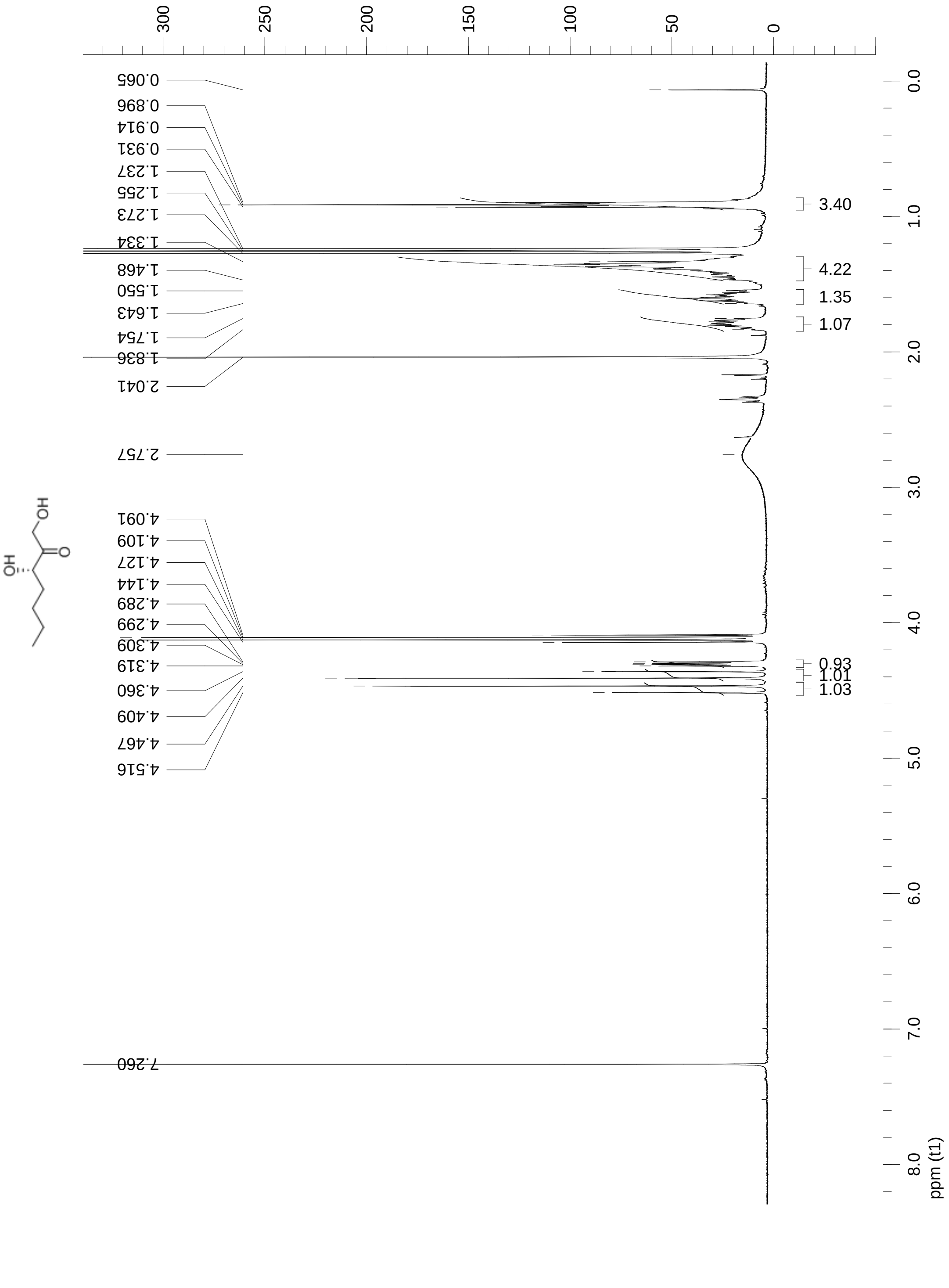

Figure S 25: **2b** 1,3-dihydroxyheptan-2-one produced enzymatically.

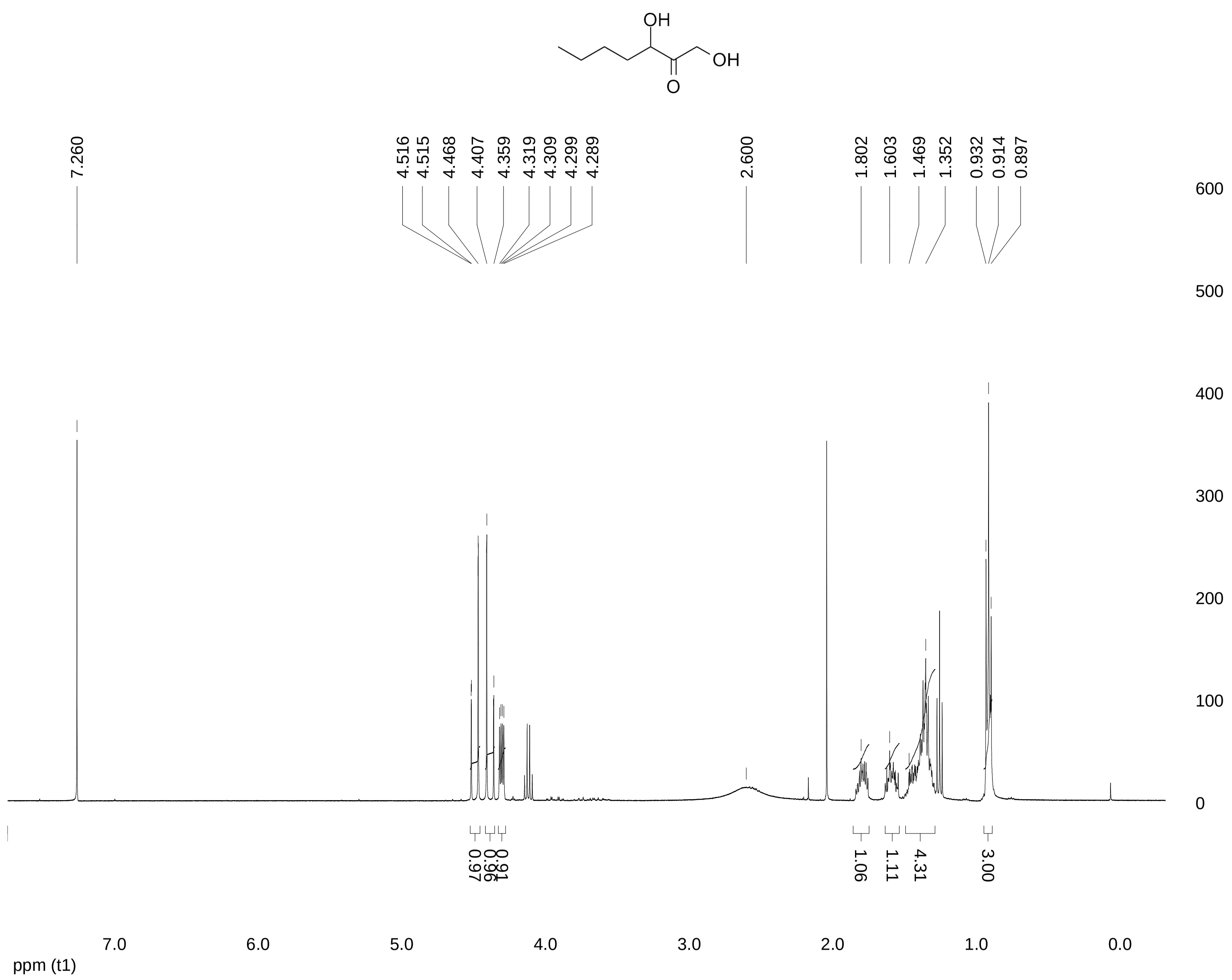

Figure S 26: Racemic **2b** 1,3-dihydroxyheptan-2-one produced by organocatalysis.

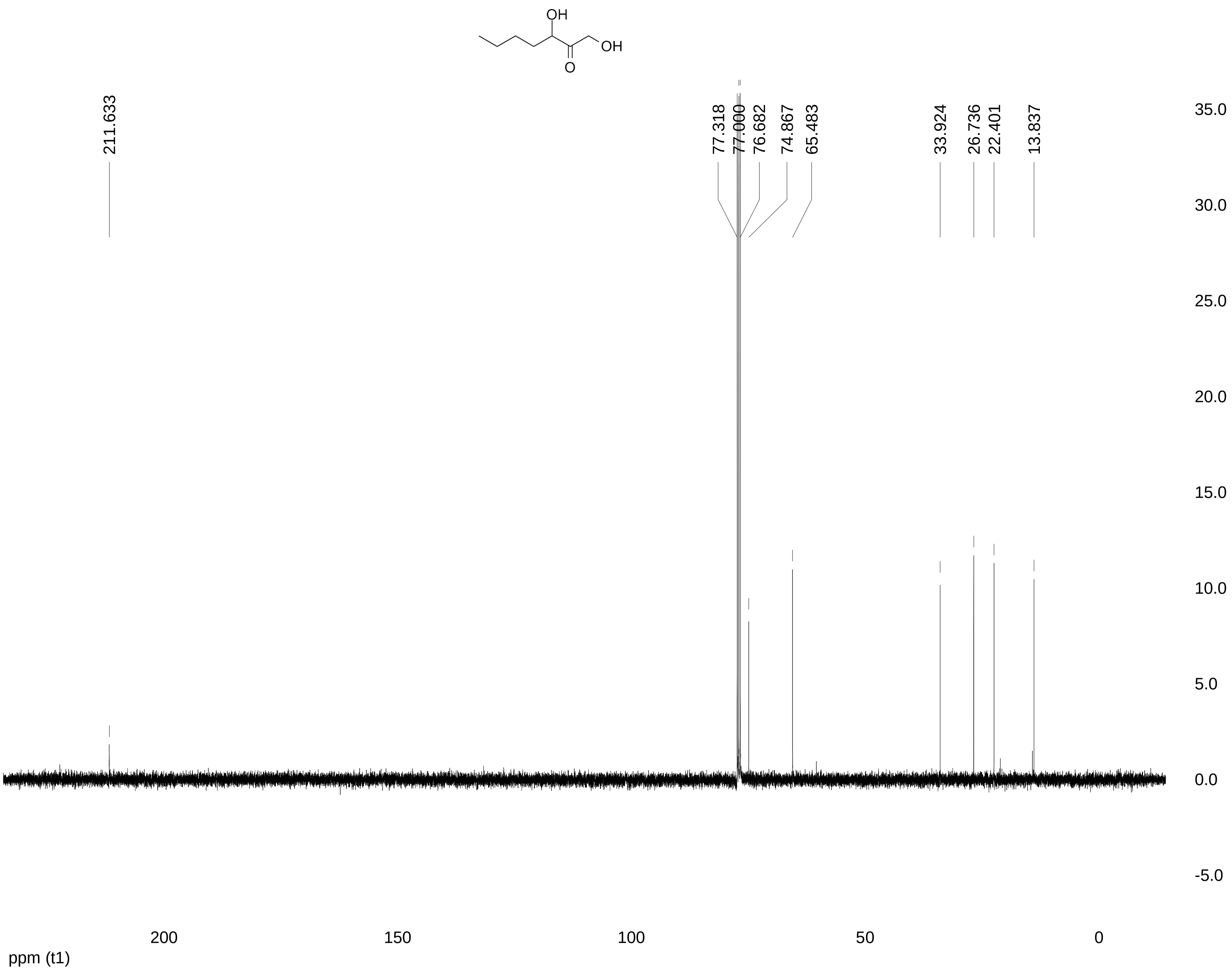

Figure S 27: Racemic **2b** 1,3-dihydroxyheptan-2-one

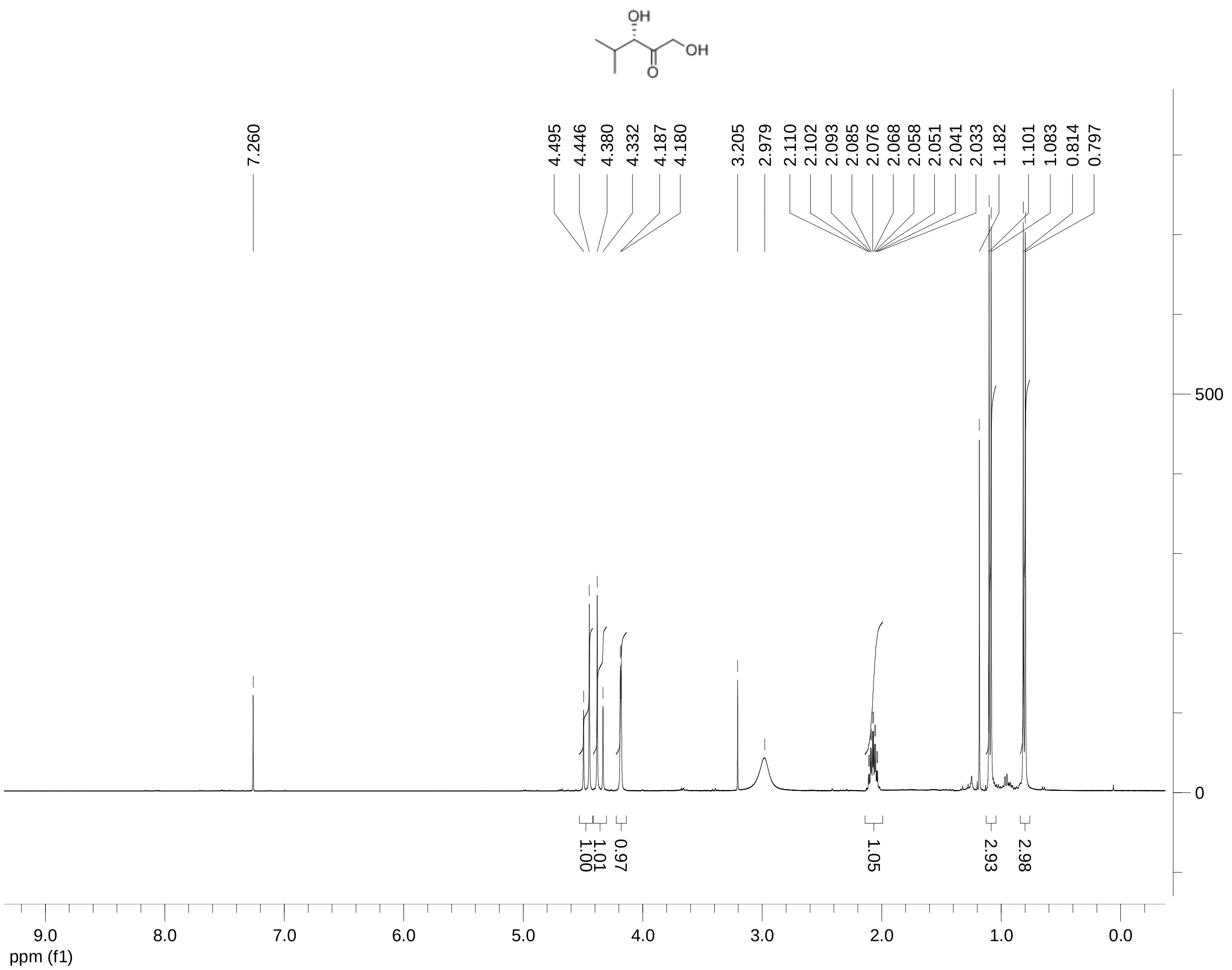

Figure S 28: **3b** 1,3-dihydroxy-4-methylbutan-2-one produced enzymatically.

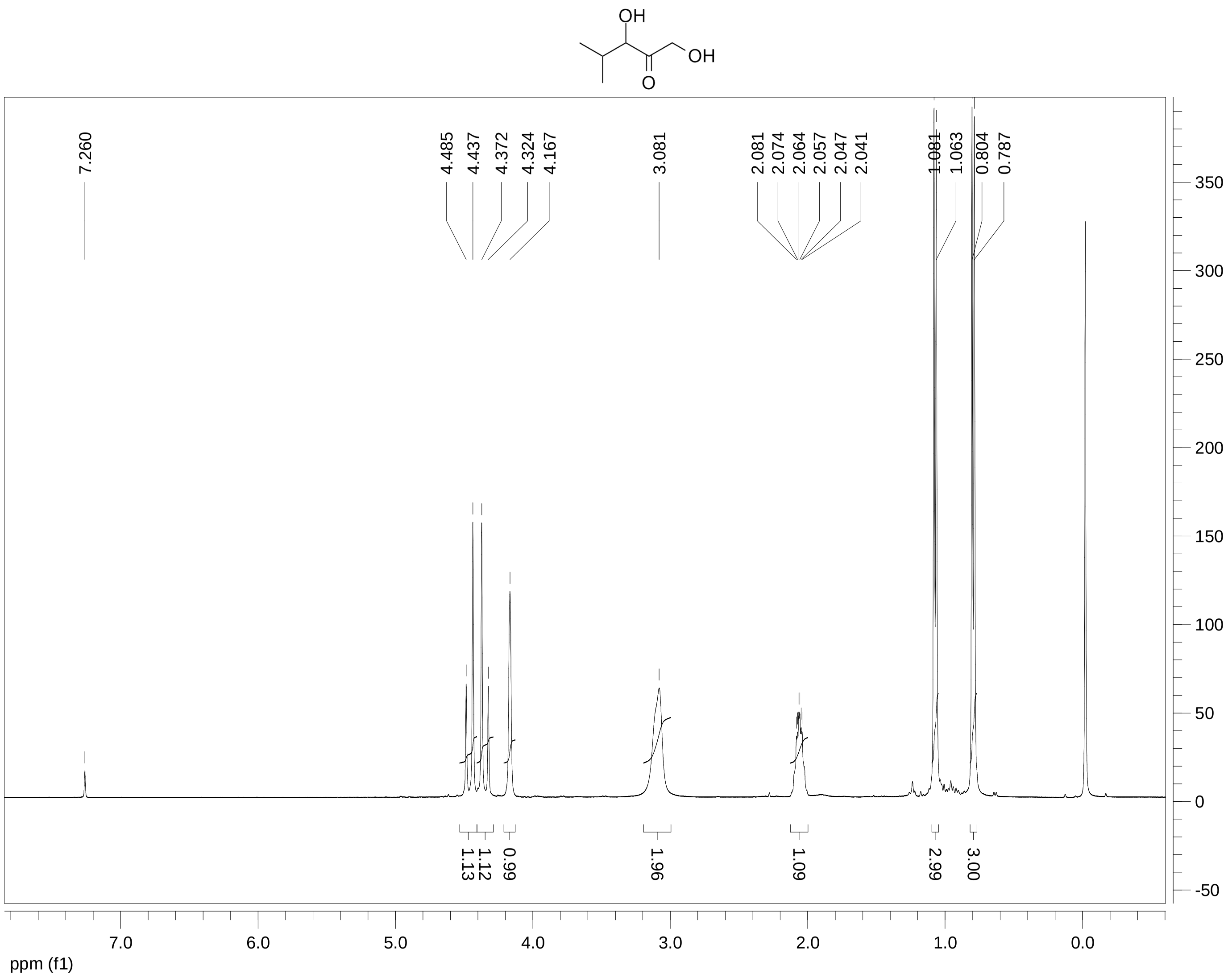

Figure S 29: Racemic **3b** 1,3-dihydroxy-4-methylbutan-2-one produced by organocatalysis.

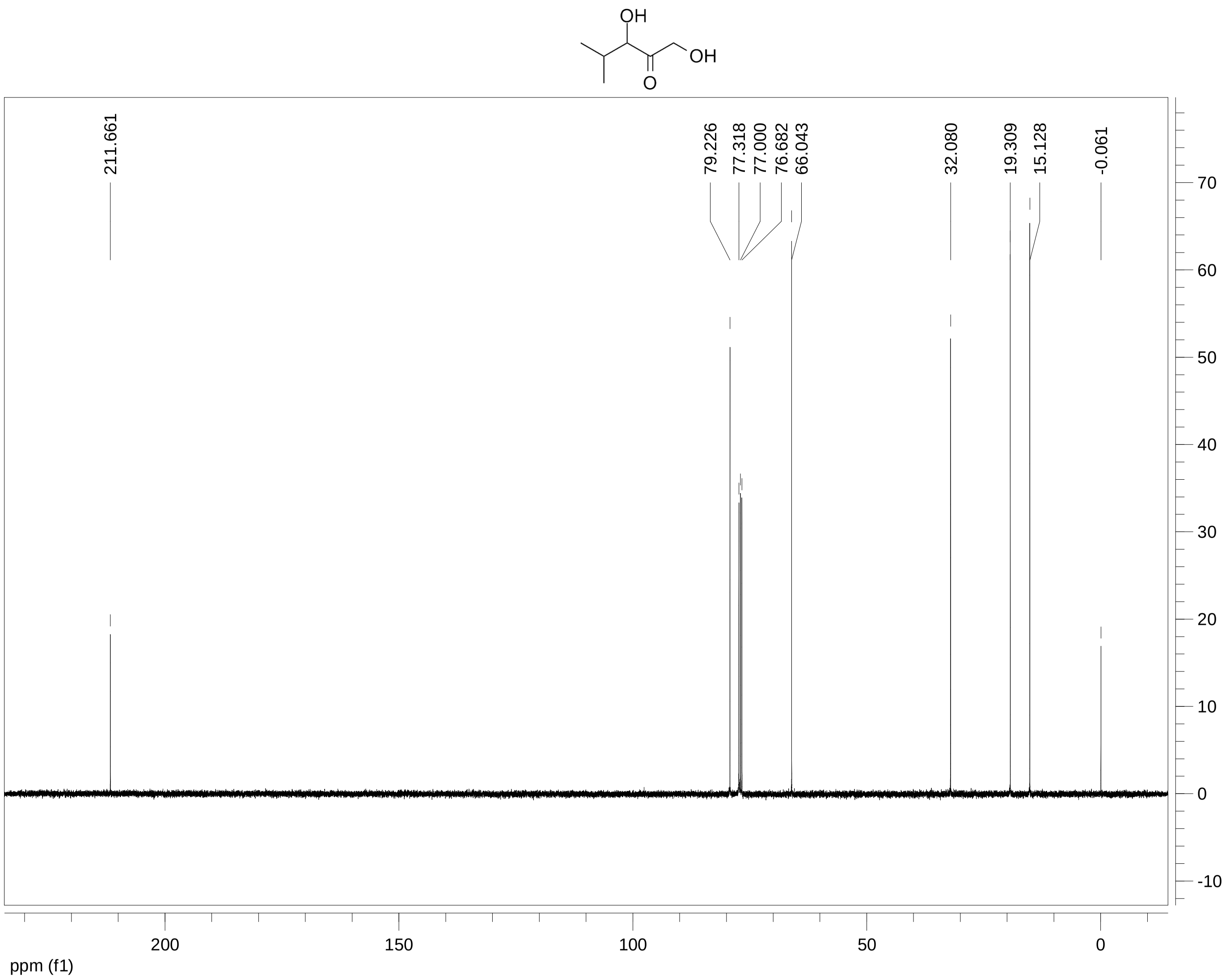

Figure S 30: Racemic **3b** 1,3-dihydroxy-4-methylbutan-2-one

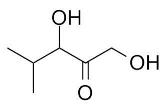

SM035

Sample Name	SM035	Pulse sequence	gHMBCAD	Temperature	25	Study owner	BOC-Marsden
Date collected	2016-03-17	Solvent	cdcl3	Spectrometer	TUD-NMR400as-vnmrs400	Operator	BOC-Marsden

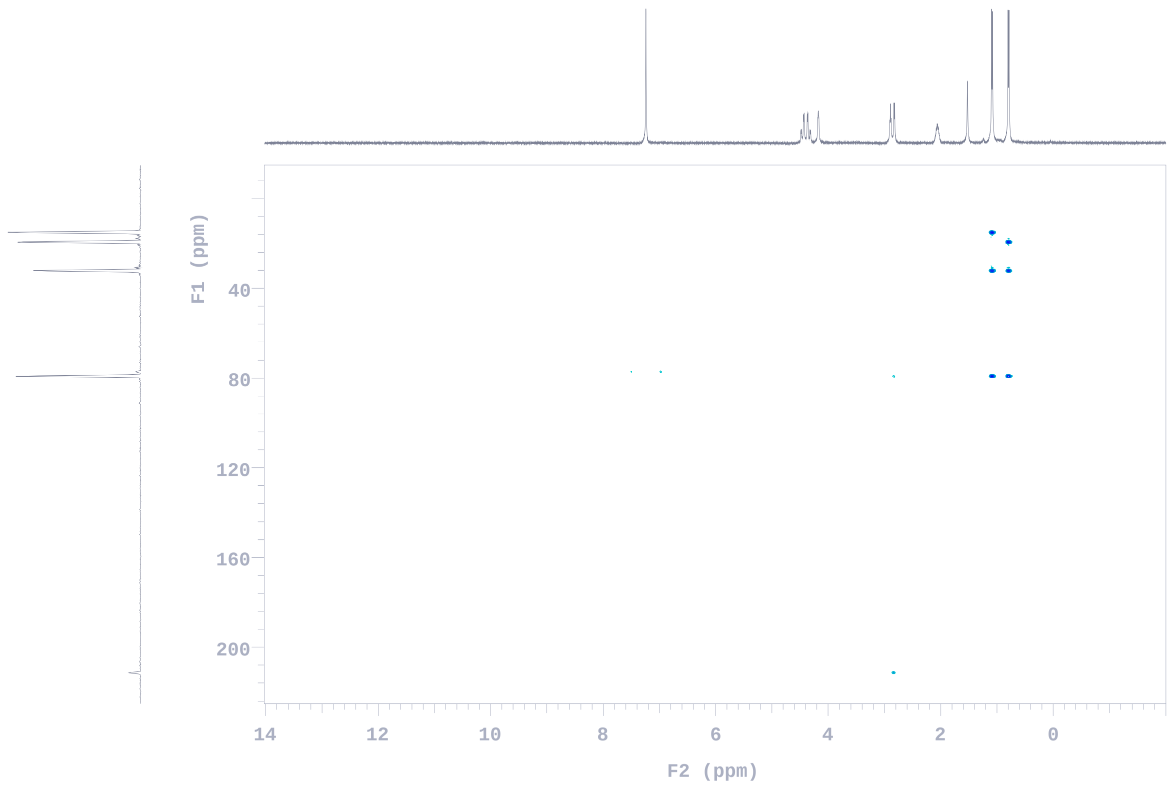

Data file /home/walk-in/vnmrsys/data/BOC-Marsden/SM03501/SM035_gHMBCAD_01.fid

Plot date 2016-03-17

Figure S 31: Racemic **3b** 1,3-dihydroxy-4-methylbutan-2-one

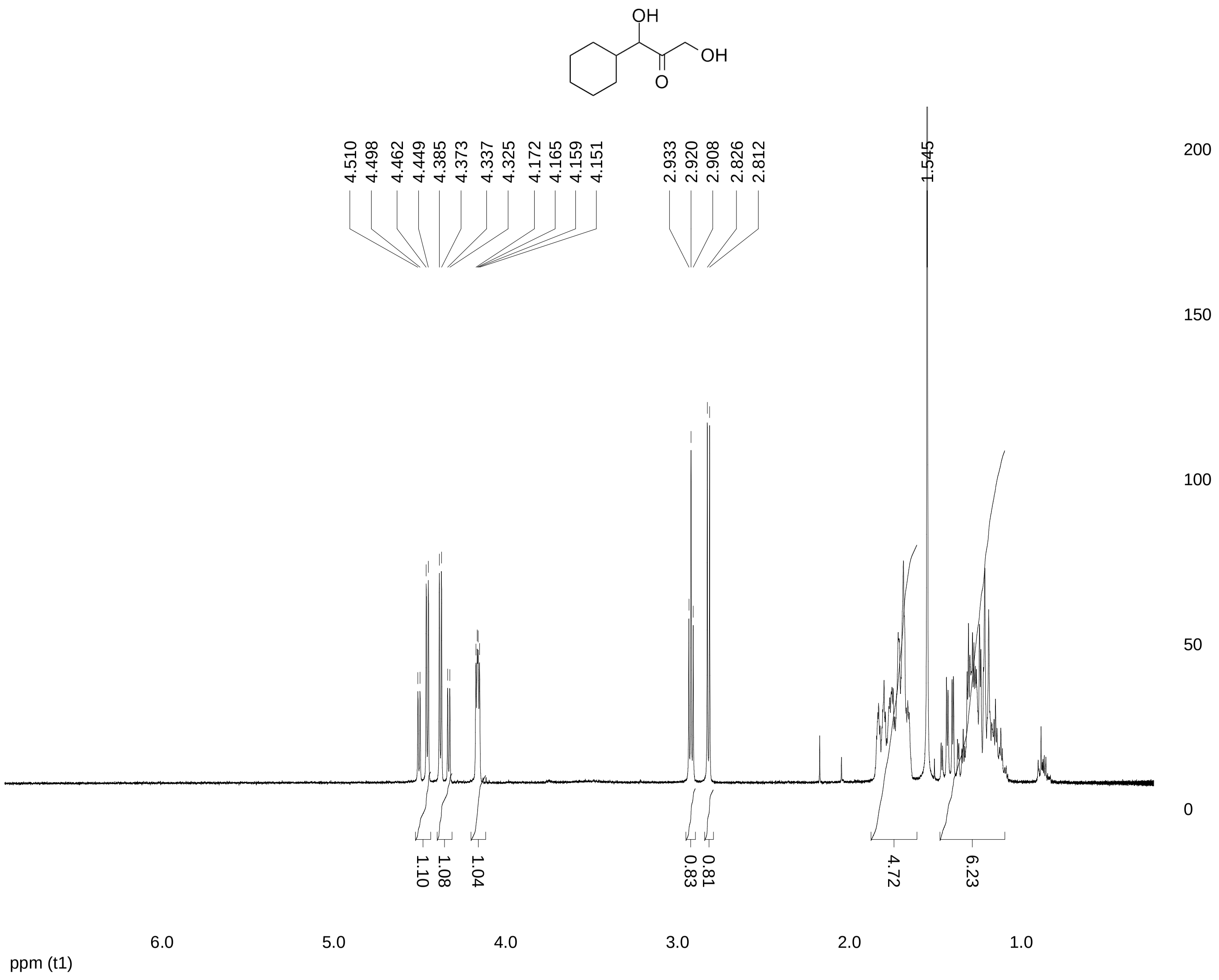

Figure S 32: Racemic **4b** 1-cyclohexyl-1,3-dihydroxypropan-2-one produced by organocatalysis.

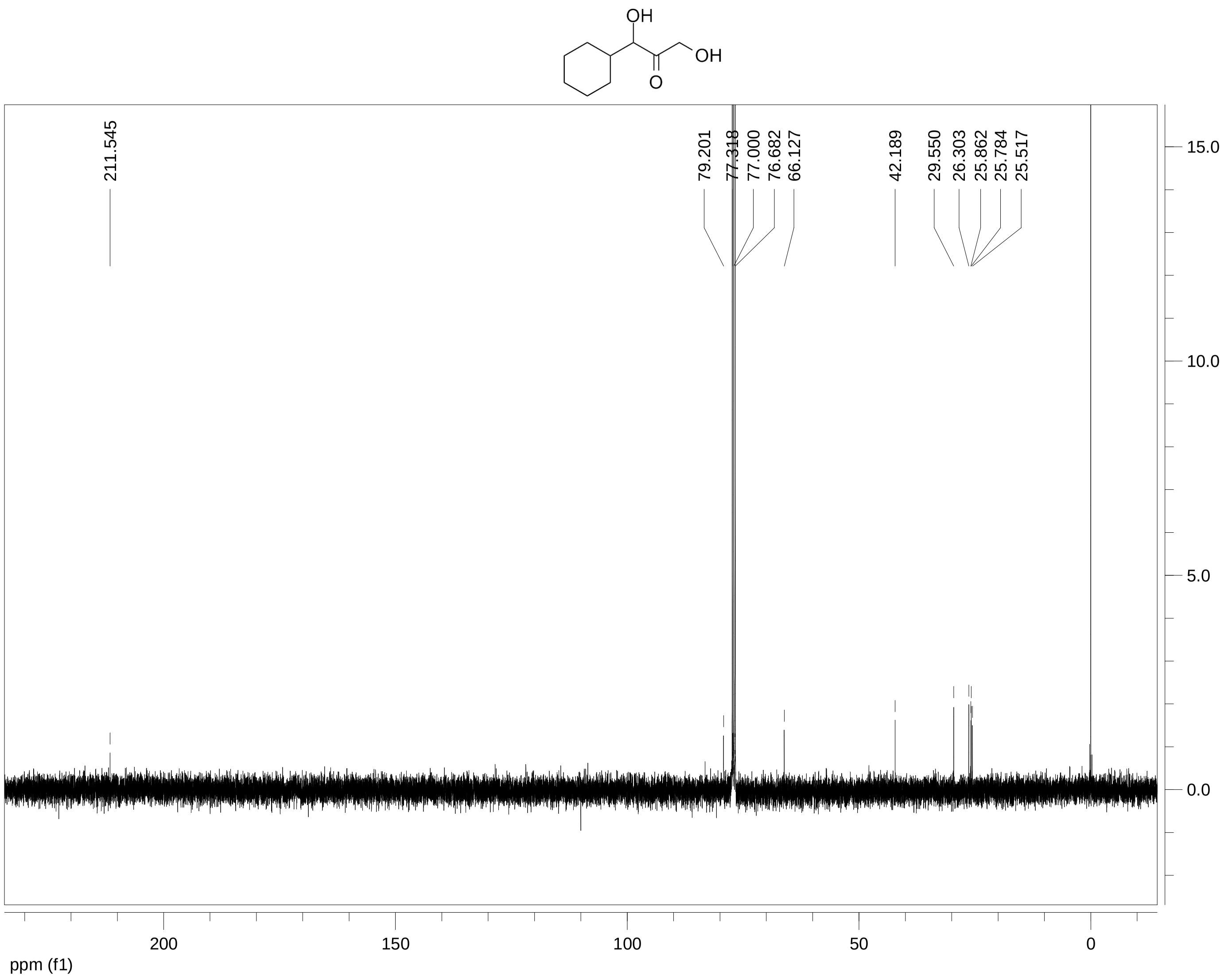

Figure S 33: Racemic **4b** 1-cyclohexyl-1,3-dihydroxypropan-2-one

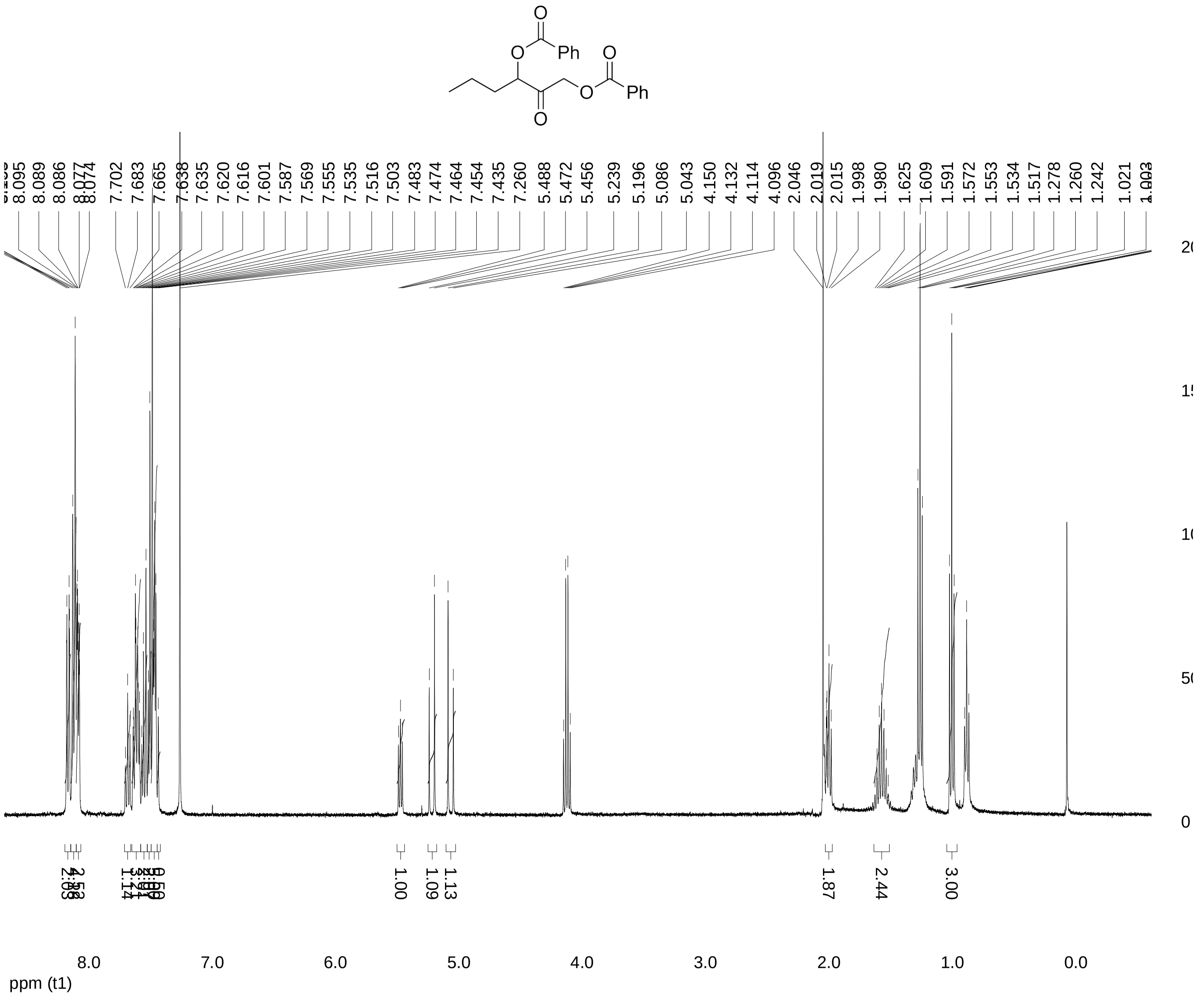

Figure S 34: Racemic **1c** 2-oxohexane-1,3-diyl dibenzoate

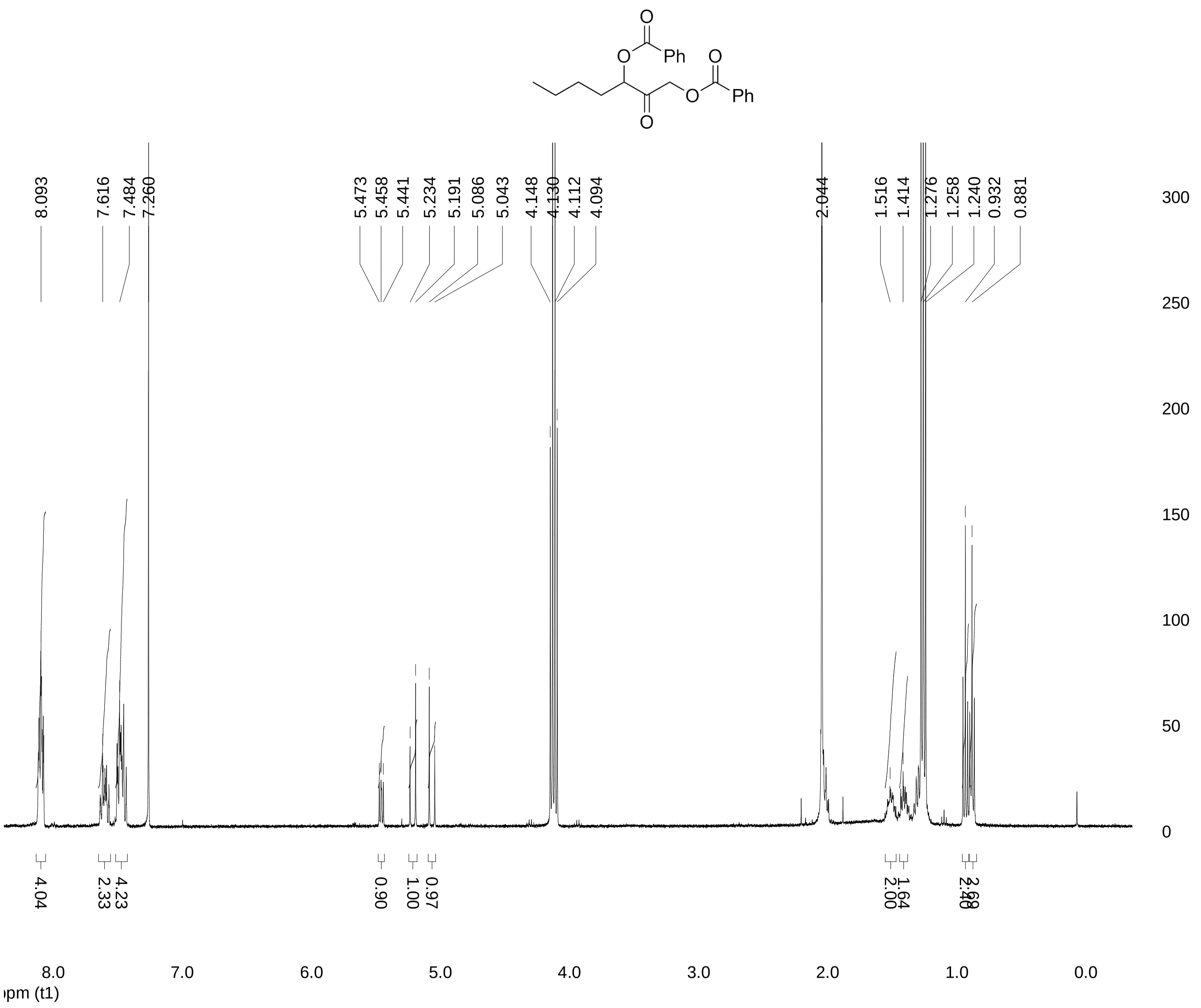

Figure S 35: Racemic **2c** 2-oxoheptane-1,3-diyl dibenzoate

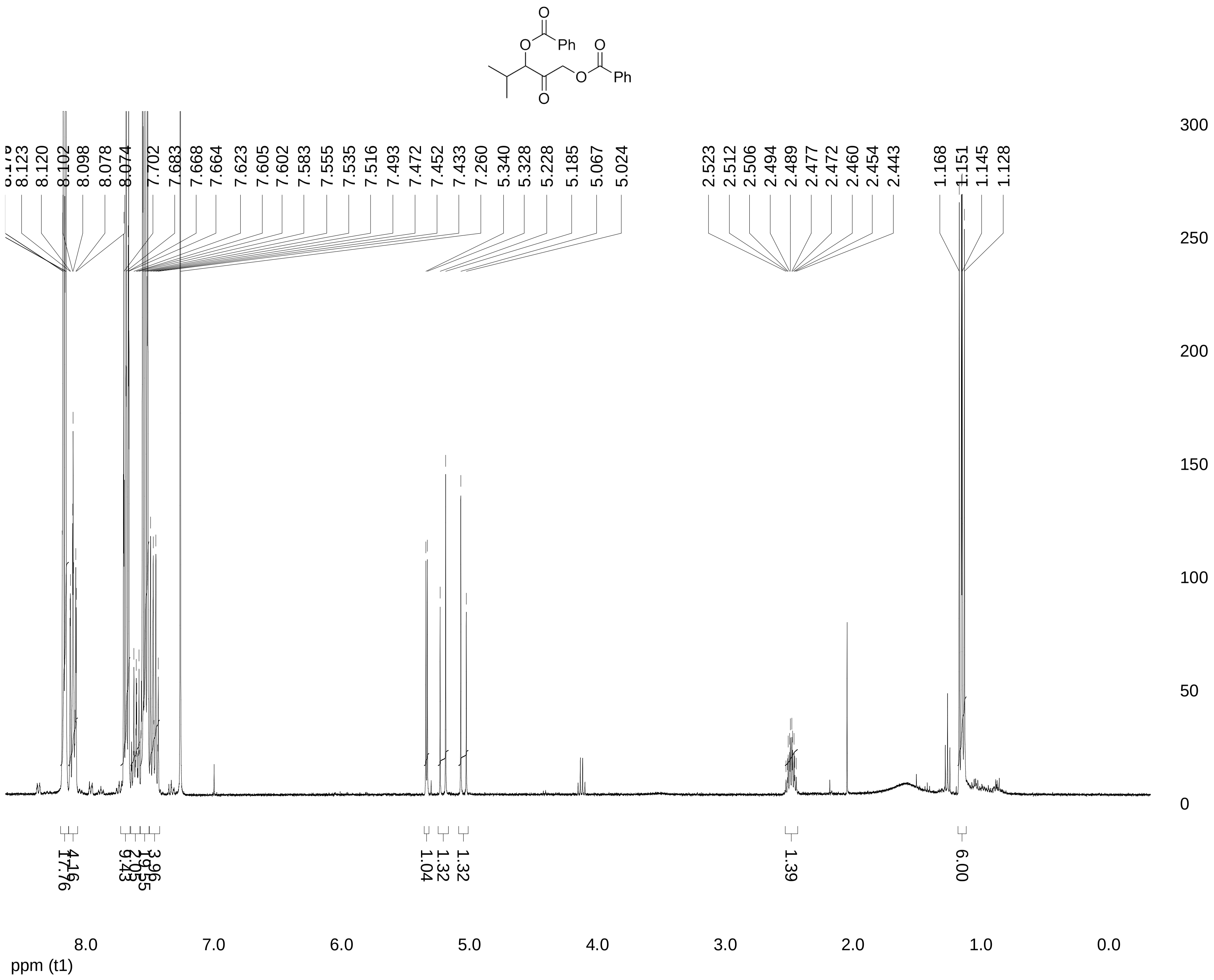

Figure S 36: Racemic **3c** 4-methyl-2-oxopentane-1,3-diyl dibenzoate

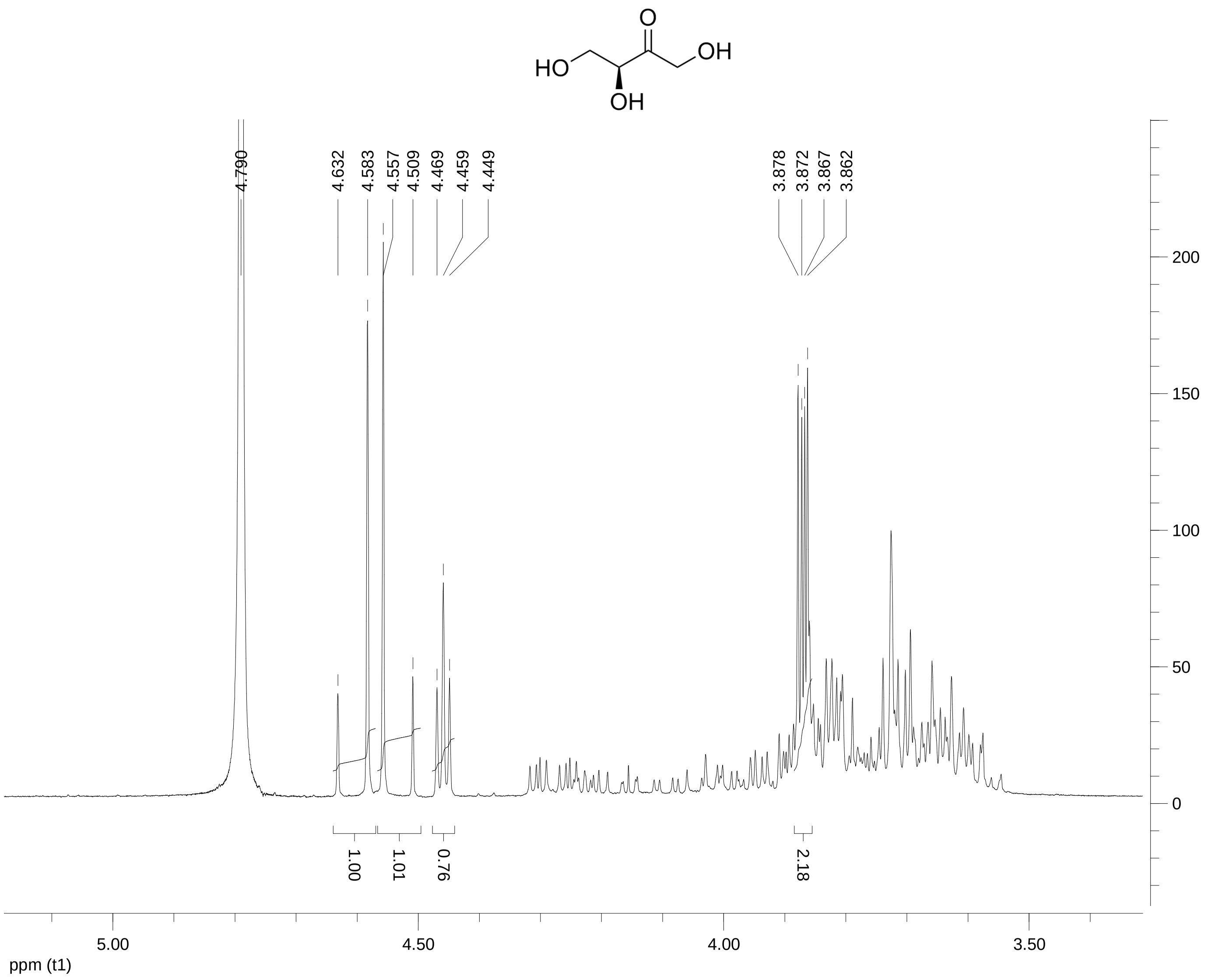

Figure S 37: Commercial L(+) erythrulose

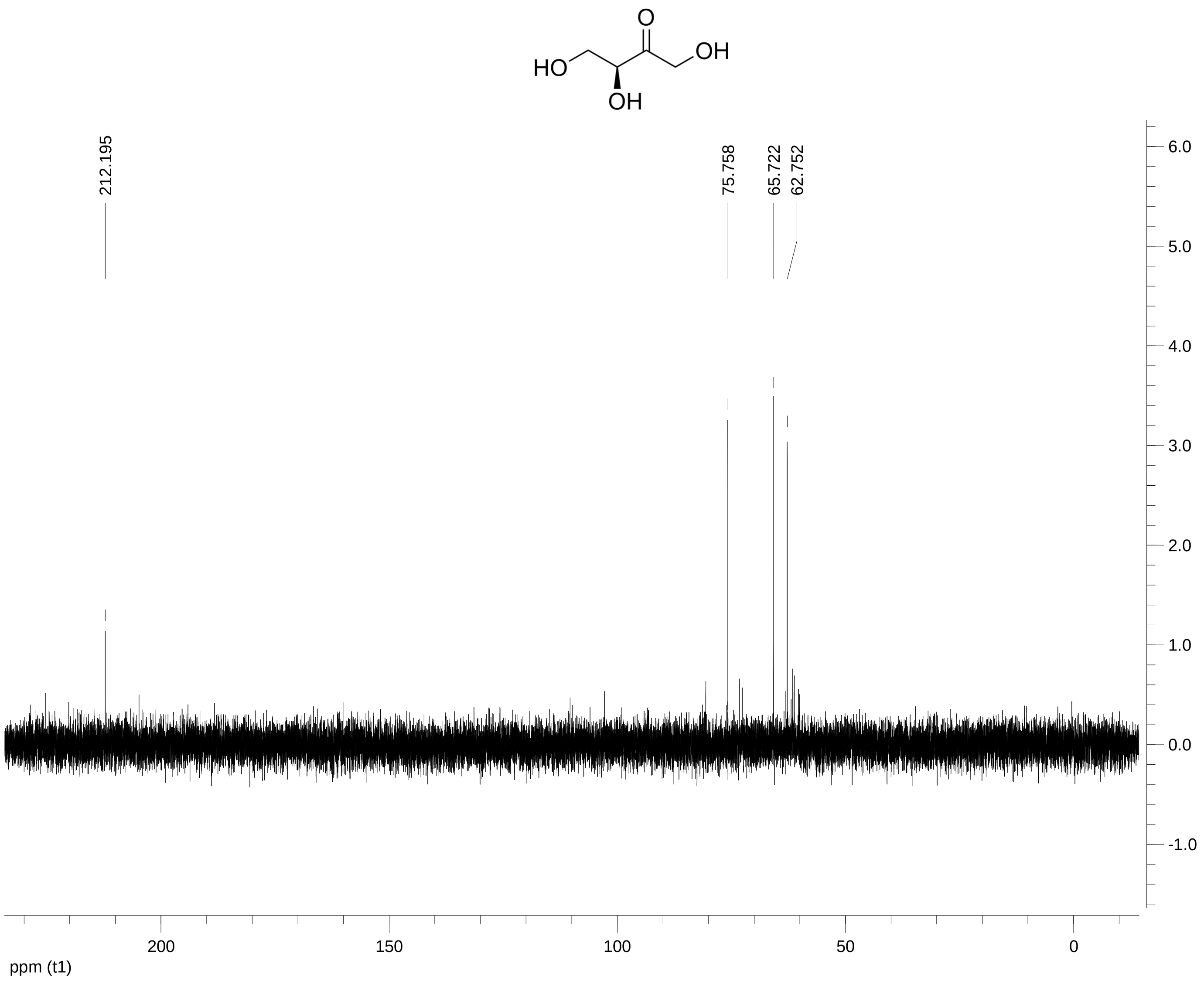

Figure S 38: Commercial L(+) erythrulose

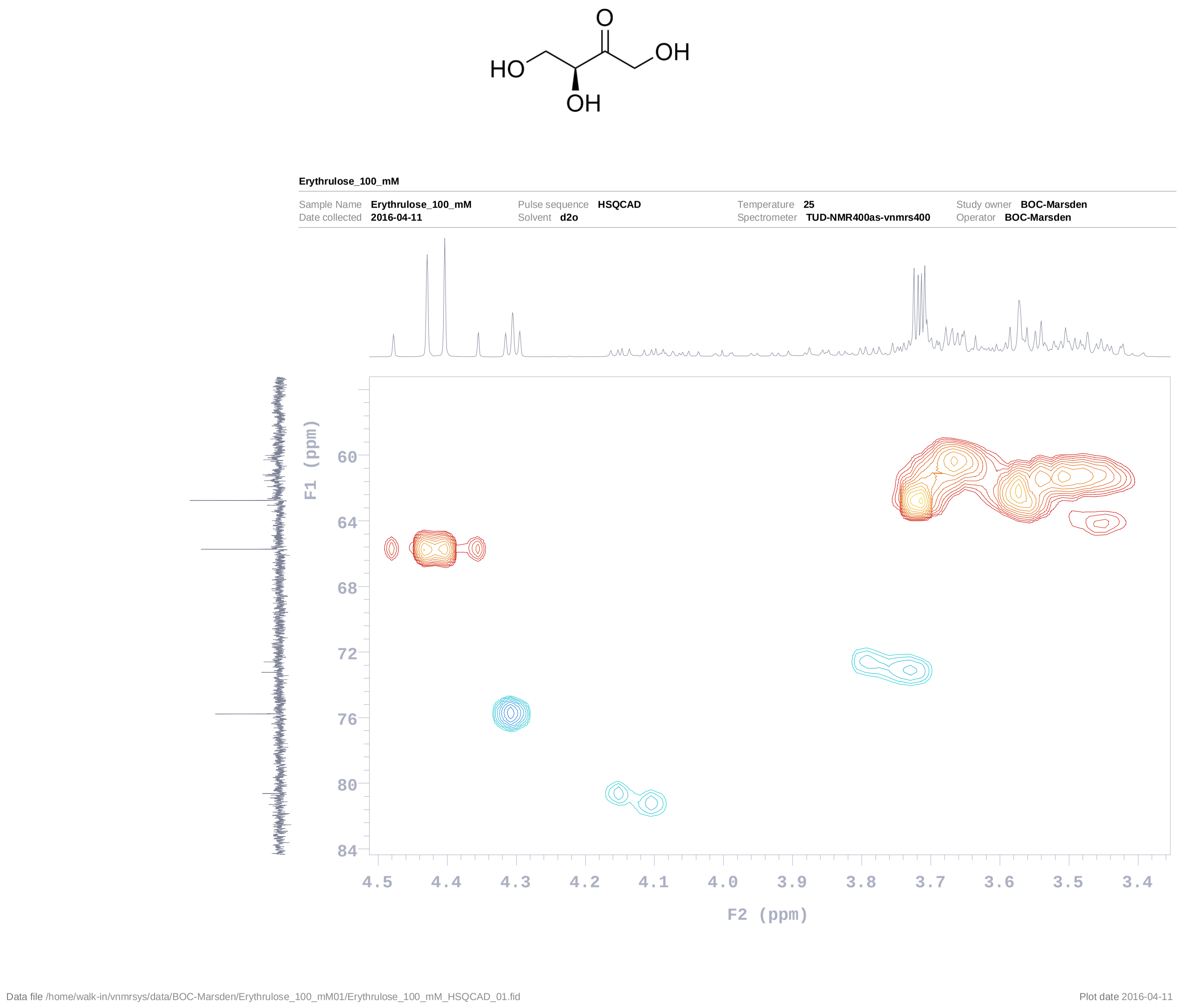

Figure S 39: L(+) erythrulose

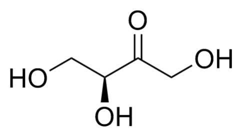

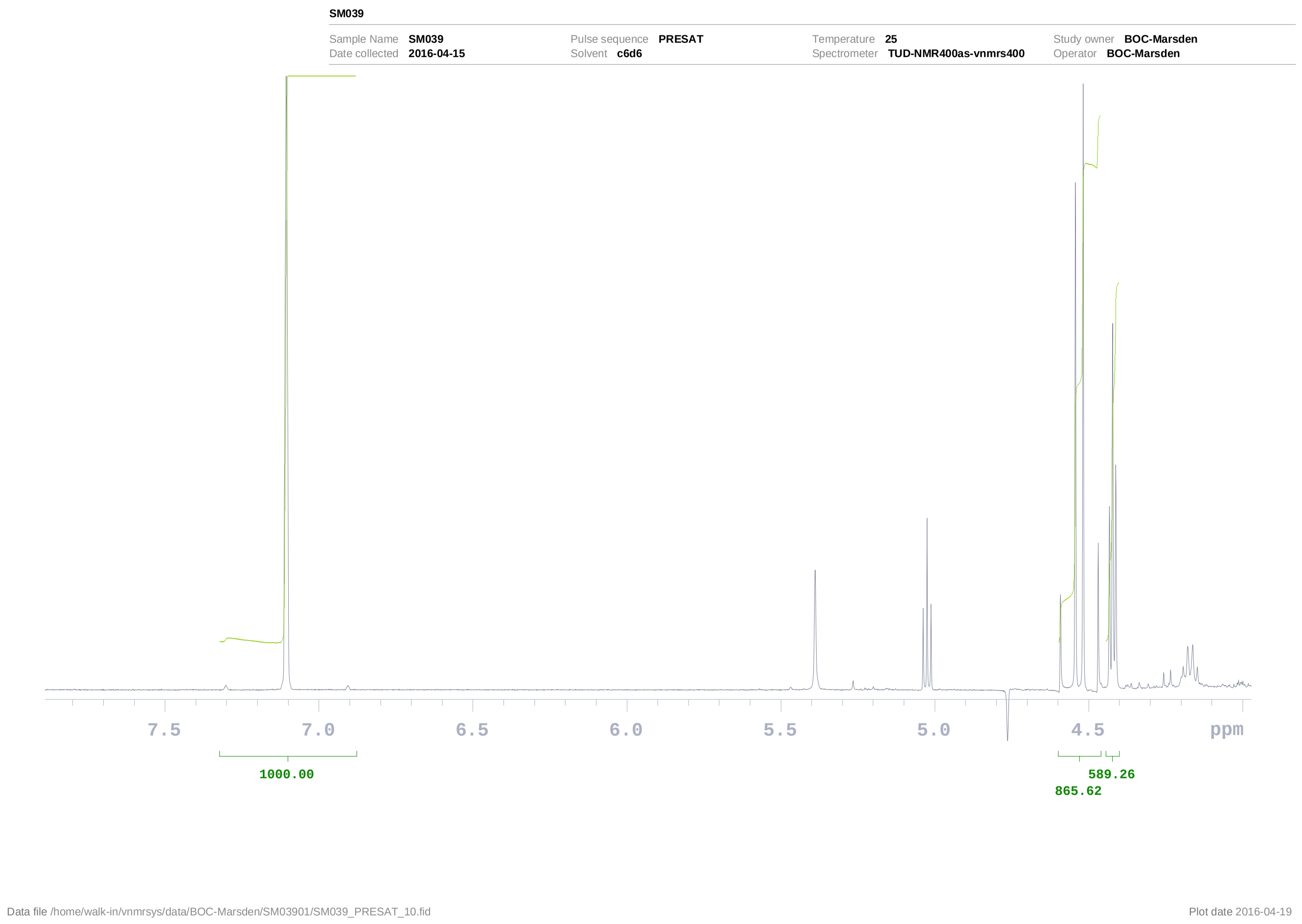

Figure S 40: Representative view of the characteristic peaks of L(+) erythrulose followed over time by NMR in aqueous KPi buffer using the PRESAT pulse sequence for suppression of the water peak.
